# The Big Bang of an epidemic: a metapopulation approach to identify the spatiotemporal origin of contagious diseases and their universal spreading pattern

**DOI:** 10.1038/s41598-025-85232-7

**Published:** 2025-02-17

**Authors:** Yazdan Babazadeh Maghsoodlo, Amin Safaeesirat, Fakhteh Ghanbarnejad

**Affiliations:** 1 Department of Applied Mathematics, University of Waterloo, Waterloo, ON N2L 3G1 Canada; 2 Department of Physics, Simon Fraser University, Burnaby, Canada; 3 Potsdam Institute for Climate Impact Research (PIK), Member of the Leibniz Association, P.O. Box 601203, 14412 Potsdam, Germany; 4 School of Technology and Architecture, SRH University of Applied Sciences Heidelberg, Campus Leipzig, Prager Str. 40, 04317 Leipzig, Germany

**Keywords:** Complex networks, Nonlinear phenomena, Infectious diseases

## Abstract

In this paper, we propose a mathematical framework that governs the evolution of epidemic dynamics, encompassing both intra-population dynamics and inter-population mobility within a meta-population network. By linearizing this dynamical system, we can identify the spatial starting point(s), namely the source(s) and the initiation time of the epidemic, which we refer to as the “Big Bang” of the epidemic. Furthermore, we introduce a novel concept of effective distance to track disease spread within the network. Our analysis reveals that the contagion geometry can be represented as a line with a universal slope, for any disease type (R0) or mobility network configuration. The mathematical derivations presented in this framework are corroborated by empirical data, including observations from the COVID-19 pandemic in Iran and the US and the H1N1 outbreak worldwide. Within this framework, to detect the Big Bang of an epidemic we require two types of data: (1) A snapshot of the active infected cases in each subpopulation during the linear phase. (2) A coarse-grained representation of inter-population mobility. Also even with access to only the first type of data, we can still demonstrate the universal contagion geometric pattern. Additionally, we can estimate errors and assess the precision of the estimations. This comprehensive approach enhances our understanding of when and where epidemics began and how they spread. It equips us with valuable insights for developing effective public health policies and mitigating the impact of infectious diseases on populations worldwide.

## Introduction

Throughout history, infectious disease outbreaks have significantly impacted human life all over the world^1,2^, causing many deaths^3^. For instance, the COVID-19 pandemic^4^, affected people of all countries^5,6^, mentally^7–9^, financially^10^, and beyond. Human mobility is a key factor in this regard^11–16^, accounting for the spatial spread of diseases, facilitating their propagation through the densely connected networks of global, national, local displacement^17–19^. The complex network of travel routes^20^ provides numerous direct and indirect pathways for disease transmission at various scales, with air travel playing a crucial role in the rapid spread of viruses including SARS-CoV-2^21–26^ at the macroscopic level, given their potential to connect distant locations^27^. This underscores the importance of taking immediate non-pharmacological interventions^28^, such as air travel restrictions^21,29^, to control disease spread. Understanding the underlying transmission mechanisms of disease at a coarse-grained level, where the mobility network can be considered as a meta-population network^30,31^–which consists of nodes, each representing a distinct patch or sub-population.– is essential for developing effective strategies to control pandemics and save lives.

More specifically, answering questions like “Where is (are) the source (sources) of an epidemic?”, “When did it begin?”, which we refer to as the *Big Bang* of the epidemic, and “How does the outbreak spread through other sub-populations?” after the Big Bang, is of paramount importance.

In recent studies, a variety of mathematical models^32^, ranging from stochastic models^33–35^, spatio-temporal spreading models^36,37^, reaction-diffusion models^38^, to agent-based models^39–43^ and network^27,44–46^ models and meta-population models^34,41,47–61^ have been developed to investigate various aspects of disease-spread phenomena. More specifically, *effective distance (ED)* can effectively address the issues stated above^13,23,62–64,64,65^.

The effective distance between two sub-populations is determined by the probability of travel along direct or indirect routes connecting them. In this context, the most likely path between two sub-populations plays a key role. In 2007, Gautreau et al.^62^ introduced an approach for calculating this effective distance. Later, in 2013, Brockmann and Helbing^13^ refined this approach, proposing an ansatz to quantify effective distance based on travel probabilities. Their method demonstrated a strong correlation between effective distance and the first arrival time of a disease, particularly when applied to the global air transportation network.

The methodology was later improved by adding the effects of all possible paths^66^, resulting in a substantial increase in the correlation between the first arrival time and ED. Also this approach has been validated with empirical data of the 2003 SARS and the 2009 H1N1 pandemics^13^. Other successful modifications on ED have also been reported^23,63,64^. For instance, Zhang *et al* introduced Country Distancing^64^, which is similar to the equivalent resistance defined for parallel resistors in electrical circuits. The idea of ED was also successfully tested for the COVID-19 pandemic on the world air traffic data^65^. This correlation between effective distance and arrival times not only suggests that effective distance can be a useful predictor of disease spread but also provides a means to trace the origin of an epidemic, helping identify the source of the outbreak^13^.

There are also many other methods developed to identify the source of an outbreak in a variety of networks such as a meta-population network or a network of individuals, and in different contexts like disease spreading^67–73^, information spreading^74–76^, food contamination^77–79^, rumors^80,80–85^, diffusion processes on networks^86–91^, etc. Typically, the aim of these studies is to identify the source of spread from a “snapshot”, for example number of infected ones, which is the state of the system after the start of the spread.

Despite the success of ED methods, there are some limitations. Some examples follow. First, their definitions often rely on intuition rather than being grounded in comprehensive mathematical models, which may hinder their clarity and interpretability, as well as impede a deep understanding of the contagious dynamics they aim to describe. Second, it is usually assumed that the disease originates from a specific location in the network (the source) and contaminates other nodes over time. However, this assumption is not necessarily true. For instance, on a country scale, the disease can reach different nodes (states or provinces) from outside the network during its spread, effectively acting as multi-sources within the country. Finally, the methods lack any correction of time to detect the beginning of the epidemic in data or any error analysis to check the validity of the spatial and temporal source estimations.

In this paper, we address these gaps by first developing a mathematical framework based on intra-population SIR model dynamics and inter-population mobility within a meta-population network. We then derive expressions to identify the source or sources of any given epidemic, as well as its starting time, given the number of infected individuals and coarse-grained mobility data at a specific time. Additionally, we propose a new definition for effective distance, which universally relates the overtaking time of nodes to their distance from the epidemic’s source. We validate this method with real-world data from the COVID-19 pandemic in Iran and the US, as well as the global H1N1 pandemic.

## Our mathematical framework

**Fig. 1:**
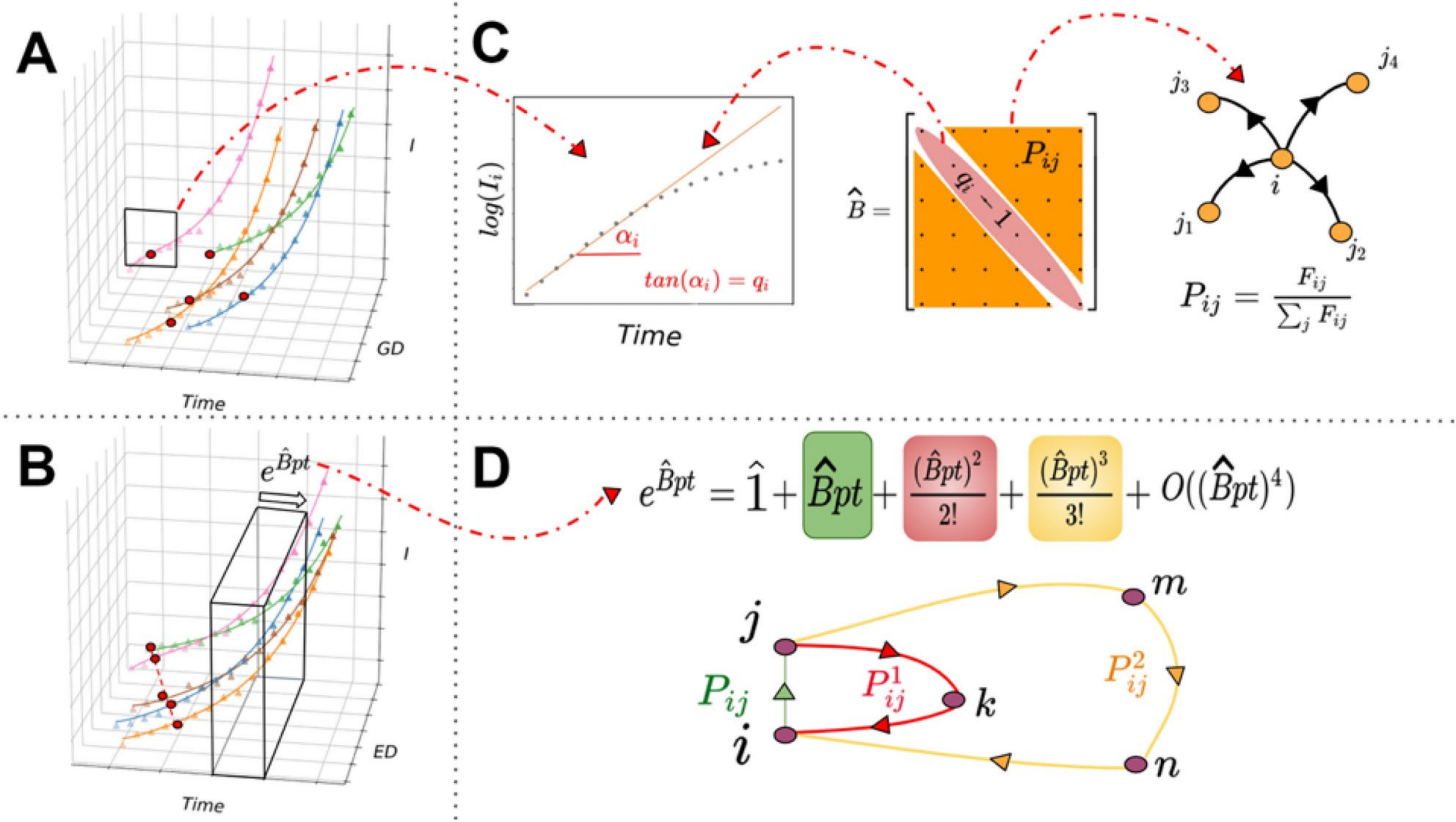
A schematic visualization of our mathematical framework. (**A**) The number of infected people in different nodes $\vec {I}(t)$ 1 in a network versus time, see data availability. The third dimension represents the geographical distance from the source in this specific network and it does not show any pattern. (**B**) The same number of infected people in different nodes in a network versus time; but this time they are plotted based on their effective distances from the source. This time a linear relation between the overtaking times and *effective distances* can be seen. The evolution of the number of infected people is given by the operator, $e^{\hat{B}pt}$, see Eq. (2). (**C**) *Center*: The matrix $\hat{B}$ is defined based on the transition probability (see S.M. 1.2) and the slope of the linear part of the SIR dynamics (see S.M. 1.4). *left*: $\log I_i$ vs time is illustrated. $q_i$ is the slope of the linear part. The behavior of $\log (I_i)$ in the early stage of the dynamic is linear and the slope of this line is $q_i$. *Right*: the transition probability is made from the flow between node *i* and j. (**D**) When $e^{Bpt}$ is expanded several terms are generated, each containing a power of matrix P. Different terms generate the probability of intermediary transitions. For example, the $\kappa _{th}$ term of the expansion corresponds to a path containing $\kappa -1$ intermediary node.

We aim to identify the ’Big Bang’ of a given epidemic’s dynamics and, specifically, to understand how the disease outbreak spreads following this initial event. In doing so, the first step is to propose our general mathematical framework which the spreading dynamics adhere to.

When studying the spread of infectious diseases at a given coarse-grained scale, the disease can be considered to spread through a meta population network. In this network, the number of infected and infectious individuals at time t and for subpopulation (node) *i*, $I_i(t)$, can be put into components of a vector we call $\vec {I}(t)$:

1 $$\begin{aligned} \vec {I}(t)=(I_{1}(t), I_{2}(t), ... , I_{n}(t)). \end{aligned}$$

Here, n is the number of nodes. In general, during epidemic dynamics, there is no specific pattern in the number of infected people across all nodes. However, typically, the number initially increases to reach a peak, followed by a subsequent decline, see Fig. 1A. In our following framework, our primary objective is to demonstrate the evolution of $\vec {I}(t)$ and subsequently discern a straightforward geometric pattern that illustrates the progression of the outbreak within nodes of the meta-population network. Therefore we show that vector $\vec {I}(t)$ evolves in time as follows, see Fig. 1B and the Supplementary Material (S.M.), Section 1 for more details:

2 $$\begin{aligned} \vec {I}(t) = e^{\hat{B}pt} \vec {I}(0). \end{aligned}$$

In this equation $\hat{B}$ is a matrix that describes the evolution of $\vec {I}(t)$ and includes $q_{i}-1$ in diagonal components, which represent the growth of the disease in each node and probability matrix $P_{ij}$ in non-diagonal components. The above equation is the solution of the following equation, assuming $S_i \approx N_i$ for the early stages of the dynamic, see S.M. 1.4 for more details:

3 $$\begin{aligned} \frac{dI_i}{dt} = [\beta _i {N_i} I_i -\gamma _i I_i ] + [p\sum _{j} P_{ji} I_j -p I_i]. \end{aligned}$$

While this equation represents the dynamic of vector $\vec {I}(t)$ and has two parts:

1. spreading within the population, namely *intra population* (First Bracket).
2. mobility between subpopulations, namely *inter population* (Second Bracket).

We use a simple Susceptible-Infected-Recovered model (SIR) with a well-mixed assumption for the first part of the equation in which $N_i$ is the population of node *i*, $\beta$ is the transmission rate, and $\gamma$ is the recovery rate. Moreover, in the second bracket, we connect all nodes, i.e. each population via a meta-population network. $P_{ji}$ in Eq. (3) represents the probability of traveling from node *i* to node *j*. We assume that the probability of traveling between all nodes is equal to the average probability of travel in the network, denoted as *p*. A detailed explanation of how the intra-population term in Equation 3 is derived can be found in S.M. 1.2 and S.M. 1.3. It’s worth noting that in this model we have not considered any immigration to the network, which is another simplifying assumption.

In Eq. (2), matrix $\hat{B}$ (We refer to this operator as Astwihad or the Black Div, who is known as the demon of death in Iranian mythology^92,93^.) keeps all of the information regarding the properties of the dynamics (Fig. 1C, center). The diagonal components ($q_i$) represent the properties of internal growth of the disease (Intra Population Dynamics). The population of infected people in each node shows exponential behavior in the early stages. So, in the plot of $\log (I)$ vs time, there is a linear pattern for each node at the beginning of the outbreak called the “linear phase”. In this study, we specifically focus on this part of the dynamics. As it can be seen in Fig. 1C, left, the slope of this line for node *i* is $q_i$, which sits into the $i_{th}$ diagonal component of matrix $\hat{B}$ (Fig. 1C, center), see more details in S.M. 1.5.

Other components of matrix $\hat{B}$ are called $P_{ij}$, which represent the probability of traveling from node *i* to *j*. For calculating the value of $P_{ij}$ we use the flow matrix, $F_{ij}$, which keeps the number of people who travel from node *i* to *j* in a specific period. (Fig. 1C, Right). Further details about probability and flow matrices are available in the S.M. 1.2.

Now we can expand the solution (Eq. 2) and write it down as:

4 $$\begin{aligned} \vec {I}(t)= \left( \hat{1} + \hat{B}pt + \frac{(\hat{B}pt)^2}{2} + ...\right) \vec {I}(0) \end{aligned}$$

This expansion contains different powers of matrix $\hat{B}$. Since $\hat{B}$ has $P_{ij}$ on its non-diagonal components, different powers of $\hat{B}$ generate different powers of matrix *P*. As matrix *P* describes the probability of transition directly from node *i* to *j*, higher powers of *P* describe the probability of indirect transition from node *i* to *j* through a specific number of intermediary nodes in between. For instance, we can define $P_{ij}^1 = \sum _k P_{ik}P_{kj}$ as the probability of mobility from node *i* to *j* through one intermediary node in the network. This is a new finding we call *Intermediate Probability*. As can be seen in Fig. 1D, the $k_{th}$ component of the expansion holds the intermediate probability of using $\kappa -1$ intermediary nodes (S.M. 1.6).

The expanded solution can get simpler even further by focusing on the early stage of the dynamic, i.e. the linear phase, and because it takes more time to transit by indirect paths via intermediate nodes. This means that in the early stages of the dynamics, as we go further into the expansion, terms become smaller. Therefore, we can simplify the dynamic by cutting the expansion up to a certain term, keeping only the first terms. This defines the time scales for our model framework. We need to be in a time range in which the assumption of linear evolution for SIR and expansion’s cut work together, or $\tau =$ ${\textit{min}}$ $\{\frac{1}{\beta - \gamma }, \frac{1}{p}\}$, which constrains the time scale.

As explained, we aim to focus on the very beginning of the spread process, when everything just started and almost no one was infected, and study the expansion of the number of infected individuals in a specific order from the start, like the idea of the *Big Bang* in cosmology. In the following section, we will introduce several algorithms to solve the challenges and find the starting time and place as well as the hidden spread mechanism of the disease.

**Fig. 2:**
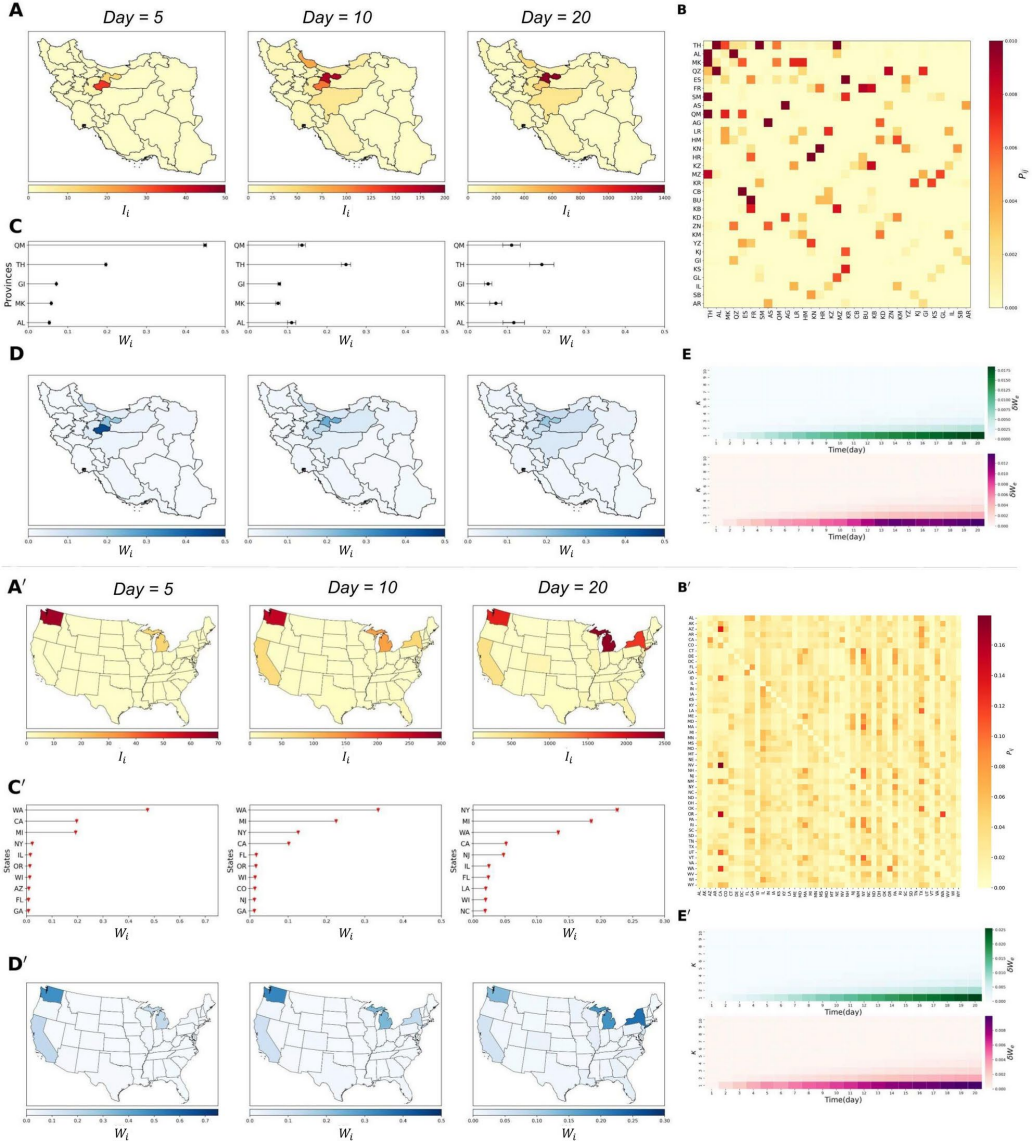
*Where did it start*? For better visibility, please zoom in. *A&A*′ Number of COVID-19 cases ($\vec {I^e}(t_e)$) across provinces/states in Iran/the US at snapshots of $t_e=$ 5, 10, 20 days from the pandemic’s start date. *B&B’* Mobility probability matrix used to calculate $W^e_i$ for Iran/the US. See S.M. Section 4 for details. *C&C’* $W^e_i$ values (Eq. 8) for provinces/states in Iran/the US, with black/red points indicating $W^e_i$ and bars showing errors, $\delta W^e_i$. We used a Taylor expansion for weights and errors (details in S.M. 2.1). *D&D’* Geographical visualization of $W^e_i$. *E&E’* Reliability plots of $\kappa$ values for the cut-off error, with $|\vec {\delta W^e}|$ shown for empirical data (top) and simulation (bottom). $\kappa =3$ and times (5, 10, 20) are highlighted in C and C’. We used $R_0 = 3$, $1/\gamma =14$ days, 31 nodes in A, B, and 50 in C, D. $P_{ij}$ and *p* were derived using empirical data $F_{ij}$ and equations in S.M. 1.2. See S.M. 5 for sensitivity analysis. The python library basemap was used to visualize the maps.

## Derivations, algorithms and results

In this section, using our mathematical framework, we introduce algorithms that reveal *where* and *when* the outbreak began and *how* it spread further using the snapshots of the disease and the flow matrix. In the first algorithm, we detect the potential sources of the disease. In the second one, we estimate the starting time of the spread, then we introduce an algorithm to illustrate a geometric pattern for the spread of the disease. There are different sources of error in the estimations such as approximating the outbreak with SIR model, inaccurate measurement of the number of infected people, and the flow matrix, which is challenging to take into account. Therefore, we only report the theoretical error caused by the cut-off in the expansion of $\exp {\hat{B}pt}$ operator, see details in S.M. Sections 2.5 and 2.8. And the role of estimating epidemiological parameters in error analysis is discussed in the supplementary text (S.M., Section 5). For the value of $R_0$ we have used the method in S.M. 1.7. Given that these algorithms rely on empirical data as input, to distinguish empirical data from theoretical variables, we denote empirical data using the subscript or superscript **e**. For instance, $\vec {I}(t)$ is a vector containing the number of infected people in our mathematical framework, and $\vec {I}^e(t)$ is the same vector but contains the empirical data of infected people coming from official reports and announcements, see S.M. Sec. 4.

### Where did it start?

Here, we aim to find the potential sources, *where* the dynamic began, having $\vec {I}^e(t)$ as empirical data and (Eq. 2) as theoretical formalism. We first develop the theoretical basis of the algorithm and then discuss how to apply it to COVID-19 data of Iran and the USA.

In a network with *n* nodes, there is a n-dimensional vector space whose i-*th* basis represents the node *i*. We define the basis ${\hat{i}}$ as

5 $$\begin{aligned} {\hat{i}} = (0,0,...,1,..0), \end{aligned}$$

in which its i-*th* component is 1 and others are zeros. Now we can expand vector $\vec {I}(t)$ in this space using these bases. The value of the component of this vector on each basis indicates the contribution of that basis to the spread of the disease. As can be seen in Eq. (2), the vector $\vec {I}(t)$ evolves in time and we have to evolve the bases in time as well, to rewind the dynamic to the origin of the time (see S.M. 2.1). Therefore, we redefine the bases as follows:

6 $$\begin{aligned} \hat{i'} = e^{\hat{B}pt} {\hat{i}}. \end{aligned}$$

Now, we define the “weight of a node” as:

7 $$\begin{aligned} W_i = \frac{\hat{i'}.\vec {I}(t)}{\sum _{\hat{i'}}{\hat{i'}.\vec {I}(t)}}, \end{aligned}$$

where “.” represents the inner product between each two vectors. The reason for this definition is to introduce $W_i$ as a number between 0 and 1 that shows the impact and contribution of the node *i* on the spreading and the initial prevalence distribution. If *t* is measured exactly from the *origin of time* of the disease, then $W_i$ represents the *spatial source of the disease*, which can be a single or multiple source. For example, for a given network if $W_i=1$, it means that node *i* was the only source of the network. For empirical data we use the vector $\vec {I^e}(t_e)$ in Eq. (7), instead of vector $\vec {I}(t)$. $t_e$ is time, measured from the officially reported temporal origin of the disease. Therefore

8 $$\begin{aligned} W^e_i = \frac{\vec {i'}.\vec {I^e}(t_e)}{\sum _{\vec {i'}}{\vec {i'}.\vec {I^e}(t_e)}} , \end{aligned}$$

It is important to note that $W^e_i$ calculation is independent of the structure of the network as there is no constraint (such as topology of the network) on the flow matrix used in the calculation. An algorithmic description of the where algorithm is provided in section 2.2.

In Fig. 2, we implement this method for the empirical COVID-19 data of Iran and the US (Panel A and A’) and illustrate the results. Also the mobility probability matrix are plotted respectively in panels B and B’. In the case of Iran (Panel C and D), we observe that the values of weights differ by implementing different snapshots. In the first scenario, Qom has the highest weight value, but when considering data from later days, Tehran (the capital province of Iran) surpasses it. Since the largest international airport in Iran (Imam Khomeini) is located between Qom and Tehran provinces, and other important nodes like Gilan and Mazandaran (which also have high values of *W*) are geographically close to this airport, we can conclude that it is more probable that the very first seed of the disease came from this airport to the country and Tehran and Qom are the most probable sources of the disease, which is consistent with official reports. For the case of the US, (C’ and D’) Washington state has the highest value of weight in the first plot, but over time, based on the values of W, and error bars, other states like Michigan, California, and New York could also be considered important nodes. So, based on this figure, our model predicts that the states of Washington, Michigan, and New York were the most probable sources of the pandemic in The US. We should mention that importation and lockdown have not been included in our analysis because we are focused on the early stage of the disease and also t=0 in this analysis regards the time when the first seed is already transported to the network (see discussion section).

The reasons for these fluctuations can be the uncertainty in empirical data (error in testing, organization error, and other sources of errors), deviation from the assumption of our model (see discussion section), and errors in calibration ($q_i$ and *p*).

To calculate the error of the Eqs. (7) and (8) we consider the value of *cut-off error* since only the first terms of the Taylor expansion of $e^{Bpt}$ is used in the calculations. If $W_i^e$ is calculated using the first $\kappa$ terms of the $e^{Bpt}$ expansion, the error of $W_i^e$ can be calculated using $\kappa$+1 term as

9 $$\begin{aligned} \delta W^e_i = \frac{(\frac{t^{\kappa +1}\hat{B}^{\kappa +1}}{\kappa !}\vec {i'}).\vec {I^e}(t_e)}{\sum _{\vec {i'}}\vec {i'} \cdot \vec {I^e}(t_e)} , \end{aligned}$$

in which “.” represents the inner product between each two vectors. The value of error depends both on $\kappa$ and the value of $t_e$. By increasing $\kappa$ or decreasing $t_e$, we expect to get a smaller value of error. To get a better idea from the value of cut-off error in a whole network, we define the *error of cut-off vector* as:

10 $$\begin{aligned} |\vec {\delta W^e}| = \frac{\sqrt{\sum _i{(\delta W^e_i)^2}}}{n}, \end{aligned}$$

which is shown versus $\kappa$ and $t_e$ for Iran (panel E) and the US (panel E’).

### When did it start?

In this section, our goal is to estimate the temporal origin of the outbreak. As we already mentioned, the real origin of time may differ from the one that is officially reported.

Assume that the source is known by the *Where* algorithm or any other method. To find the temporal origin we compare the estimated ($\vec {I}(t)$) and reported ($\vec {I^e}(t_e)$) number of infected people at the time *t* and $t_e$, respectively. We find the temporal origin so that it minimizes the mean squared error (MSE) between $\vec {I}(t)$ and $\vec {I^e}(t_e)$,

11 $$\begin{aligned} \Delta _i (t)=\frac{\sum _{j=1}^n (I_j(t) - I_j^e(t_e))^2}{n}. \end{aligned}$$

**Fig. 3:**
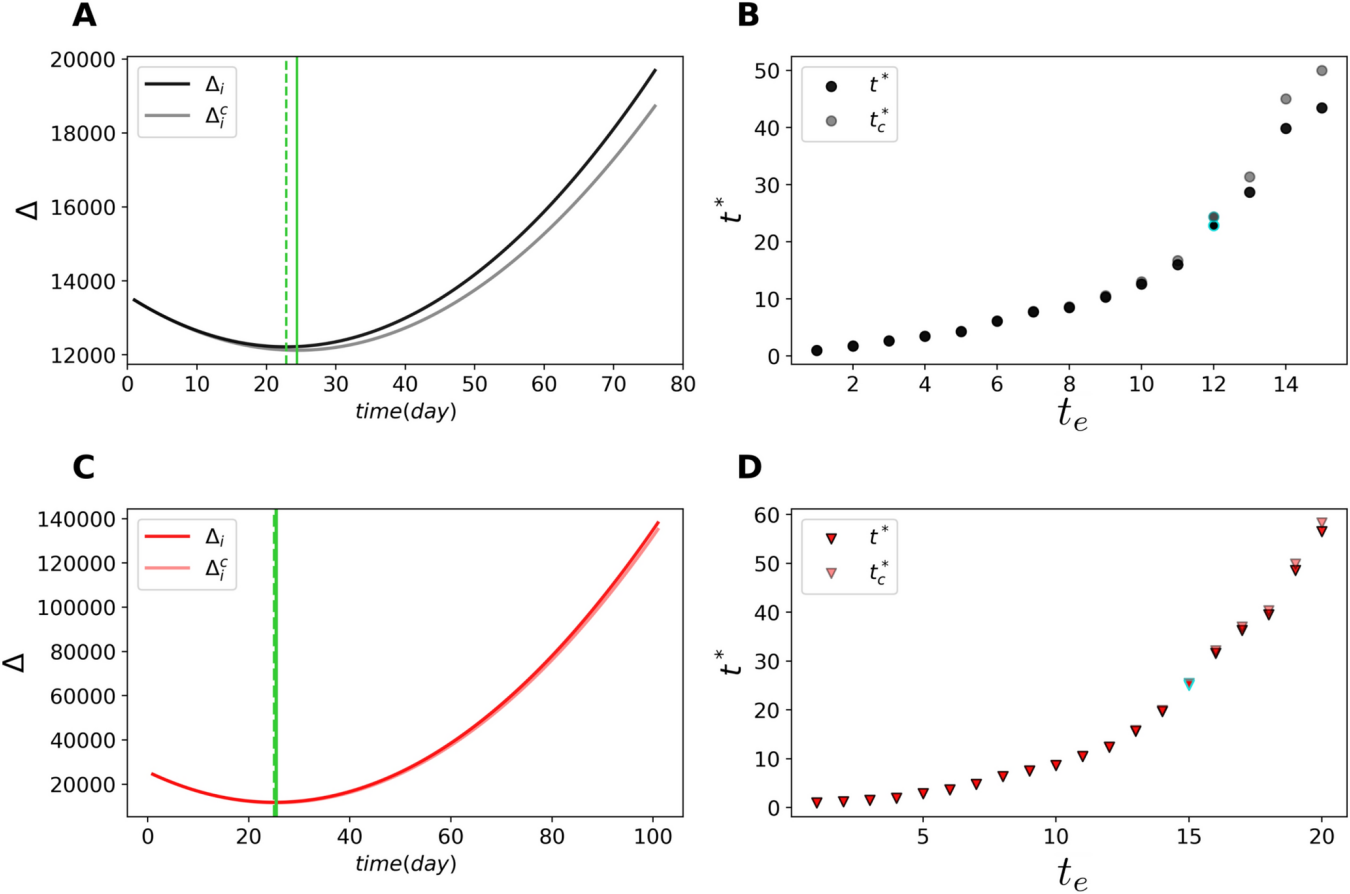
*When did it start*? (**A**) The value of MSE (Eq. 11) is illustrated versus time (the black curve) for Qom province using a snapshot for the $12_{th}$ day of the COVID-19 pandemic in Iran. As shown, it has a minimum at ($t_i^*$) (Eq. 66). When the third term of the expansion is added to the calculation, $\Delta _i$ transforms to the gray curve ($\Delta _i^c$), with the minimum shifted approximately two days. (**B**) The estimated origin of time ($t^*_i$) versus the date of the snapshot ($t_e$) for Iran, considering Qom as the origin node. The points highlighted in green show the used snapshot in panel (**A**). (**C**) The value of MSE (Eq. 11) is illustrated versus time (the red curve) for the Washington state using a snapshot for the $15_{th}$ day of the COVID-19 pandemic in *the US*. It has a minimum at ($t_i^*$) (Eq. 66). When the third term of the expansion is added to the calculation, $\Delta _i$ transforms to the pink curve ($\Delta _i^c$), which approximately lies on the red curve. (**D**) The estimated origin of time ($t^*_i$) versus the date of the snapshot ($t_e$) for the US, considering Washington as the origin node. The points highlighted in green show the used snapshot in panel (**C**). In this analysis, we used the same setup as in Fig. 2; Please refer to the S. M. Section 4 for more details regarding the data.

To estimate the number of infected people, $\vec {I}(t)$, we assume the source is the node *i* found by the *Where* algorithm, Eq. (11) has a unique minimum at time $t^*_i$ (see S.M. 2.3 and (Fig. 3)) :

12 $$\begin{aligned} t^*_i= \frac{1}{i_0} \frac{-\eta (i_0 - I_i^e(t_e)) + p \sum _j(P_{ij}I_{j}^e(t_e))}{\eta ^2 + p^2 \sum _j P_{ij}^2}, \end{aligned}$$

in which $\eta = (\beta _i N_i - \gamma _i - p)$, in which $\beta$ is the transmission rate, $N_i$ is the population of node *i*, $\gamma$ is the recovery rate and *p* is the average probability of travelling., $i_0$ is the initial number of infected people in the source, $P_{ij}$ is the probability matrix. For the error, we consider the cut-off error by adding the third term of the Taylor expansion as the error-making term to our calculation which shows how the predictions degrade (S.M. 2.5). An algorithmic description of the when algorithm is provided in section 8.4.

Figure 3 demonstrates the result of our algorithm applied to the empirical data of Iran and the US. In panel A and C, $\Delta$ (Eq. 11) is shown versus time for Iran and the US, respectively. A minimum exists in both cases, as we showed in Eq. (66). By adding the third term of the Taylor expansion (Eq. 4) to the calculation, the corrected MSE (specified with different colors) shifts to a new curve and the minimum moves a bit (around two days for Iran and less than a day for the US). To understand the accuracy of the algorithm, we illustrate $t^*_i$ versus $t_e$ for Iran (panel B) and the US (panel D) using snapshots from various days. A linear behavior can be observed up to a certain point in both cases, which indicates the linear range of the dynamics. It is worth noting again that $t^*_i$ describes the starting time of the disease from the epidemiological point of view, while $t_e$ refers to the starting time the official reports claim. So, a difference between these two origins of time is expected. By utilizing these specific snapshots, the onset of the COVID-19 pandemic is estimated to be 8 February 2020 for Iran and 12 February 2020 for the US, marking the commencement of the widespread outbreak in Iran and the US.

### How does it spread? The universal pattern of any outbreaks

In previous sections, we estimated the origin of the disease, trying to answer when and where it began. In this section, we aim to illustrate the simple geometric patterns behind the dynamics.

For the first step, let us define the *overtaking time* in our mathematical formalism. This is the time when enough number of infected passengers arrive in a susceptible node so that we can consider this node as infected. In other words, when the intra-population spreading in this node (the first bracket in Eq. 3) equals the inter-population spreading (the second bracket in Eq. 3), which means

13 $$\begin{aligned} (N_j\beta _j - \gamma _j)I_j = p \left( \sum P_{kj}I_k - I_j\right) . \end{aligned}$$

Using the above condition and the dynamic of our model given by Eq. (4), one can show that the overtaking time is (see S.M. Sections 2.6, 2.7 and 2.8 for more details)

14 $$\begin{aligned} t_{O}^{j} = \frac{1}{p} \frac{1}{(2+q_j-q_i)-\frac{P_{ij}^1}{P_{ij}}}, \end{aligned}$$

in which the overtaking time, $t_O^j$, has been calculated for the node j, given the single source, node *i*, in the network.

To detect a simple *geometric pattern* of the spread dynamic, as shown in Fig. 1.b, we define an *effective distance* between a non-origin node *j* and the single origin node *i* so that there is a *linear relation* between overtaking time (Eq. 87). Therefore, our effective distance is defined as

15 $$\begin{aligned} D_{ij} = \frac{1}{(2+q_j-q_i)-\frac{P_{ij}^1}{P_{ij}}}, \end{aligned}$$

where

16 $$\begin{aligned} D_{ij} = pt_O^{j}. \end{aligned}$$

Our innovative approach to defining effective distance distinguishes itself from previous methods^13,62,66^. While maintaining a similar geometric pattern of spread, our method uniquely utilizes overtaking time rather than arrival time, which is when the first infected patient arrives. Remarkably, we reveal a *universal* behavior, characterized by a consistent *slope of one* when plotting $D_{ij}$ against $pt^j_O$, regardless of network or disease characteristics. In the above equation, *p* represents the inter-population speed of the disease spread among the nodes in a network.

Our proposed effective distance becomes simpler for some special cases. For example, if all nodes have the same value of q, the effective distance simplifies as

17 $$\begin{aligned} D_{ij} = \frac{1}{2-\frac{P_{ij}^1}{P_{ij}}}, \end{aligned}$$

which is independent of the disease properties and only depends on the the travel flow. The defined effective distance only includes those nodes that satisfy $\frac{P_{ij}^1}{P_{ij}}<2$ as effective distance and the overtaking times should be positive for the non-origin nodes. When this condition is not met, an alternative approach may be necessary in order to include other nodes. Further studies could investigate such adaptations.

**Fig. 4:**
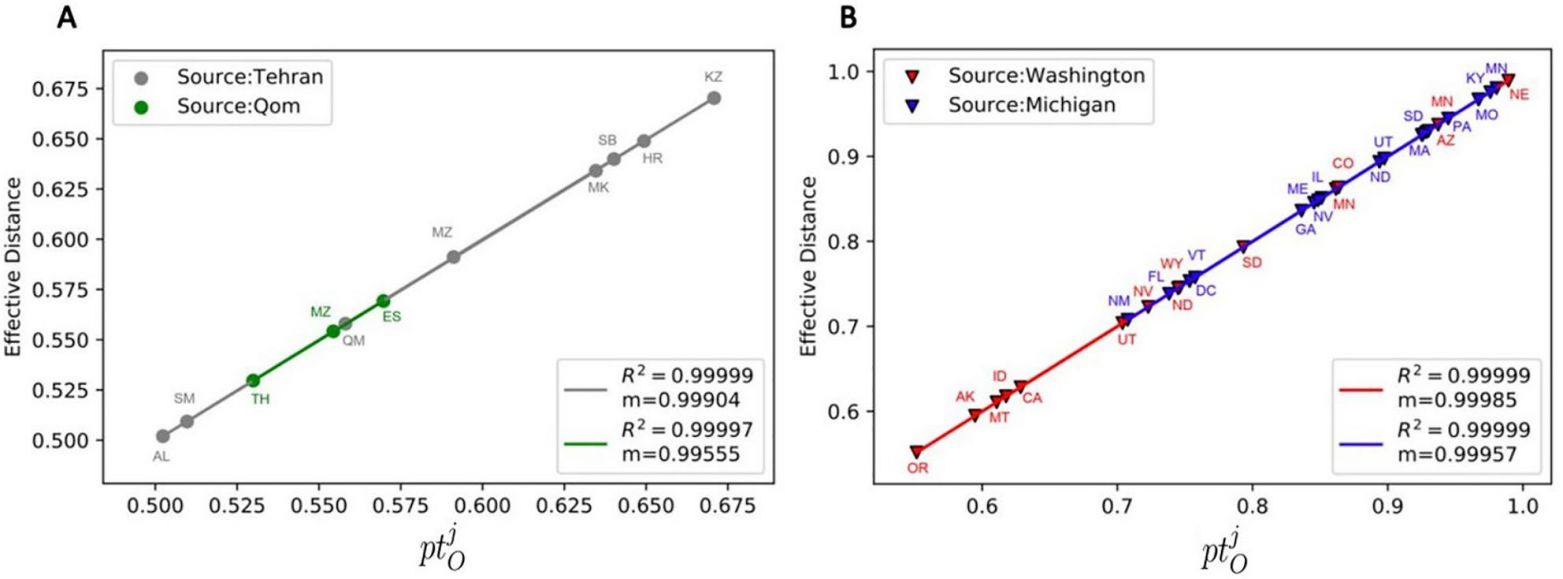
Effective distance vs overtaking time (simulation result): The effective distances are illustrated versus $pt^j_O$ (Eq. 88) for the empirical mobility data of Iran (Panel **A**) and the US (Panel **B**). Each panel represents two scenarios: we put the initial seed of the disease on a different node in each scenario. These two nodes are selected from the nodes with the higher chance of being the original COVID-19 source in Iran and the US based on the *Where* algorithm results. In this analysis, we used the same setup as in Fig. 2. In panel A, Gray/Green nodes represent the results of the simulation for Tehran/Qom, respectively as the source nodes. Also, the gray and Green lines are the best-fitted lines to the data, with their slope and regression shown in the legend. In Panel B, Red/Blue nodes represent the simulation results for Washington/Michigan as source nodes, with the Red and Blue lines showing the best-fitted lines to the data. Please refer to the S. M. Section 4 for more details regarding the mobility data.

Figure 4 shows the result of the effective distance analysis in two panels. In both panels, the spreading of the disease has been simulated with the SIR model for meta-population networks using the empirical mobility data of Iran (panel A) and the empirical mobility network of the US (panel B). In each panel, the simulation has been repeated twice (Green and Gray in the left panel, Blue and Red in the right panel), each time with a different source node. The *Where* algorithm is used in the specific choice of the source nodes. As shown, there is a linear relation between the defined effective distance and *pt*, with the universal slope of one. Changing the source node does not change the linear relation and the value of the slope.

Implementing effective distance analysis with empirical data can pose several challenges. Some of these are outlined below. First, what is reported as the arrival time in official data is not necessarily the same as what we defined as overtaking time in Eq. (87), even though they are close. Second, measuring the exact value of the mobility probability matrix is difficult, especially due to the intervention policy in each region at the beginning. Finally, the initial number of infected people ($i_0$) is not necessarily known.

It is possible to overcome the challenges stated above by estimating the effective distance of the node *j* from the source using the number of infected people in that node ($I^e_j(t_e)$). Using the mathematical framework, one can show that $I^e_j(t_e)$ can be estimated by a parabola in a short enough time after the arrival of the disease to the node.

18 $$\begin{aligned} I_j=Q_j+A_j T + B_j T^2 \end{aligned}$$

One can show that using the coefficients of this equation and the mathematical framework of this model, we can rewrite the effective distance as:

19 $$\begin{aligned} D_{ij} = \frac{1}{q_j-\frac{B_j}{A_j}} \end{aligned}$$

Also estimate the overtaking time by assuming $I_j = 0$ and solve the equation for T:

20 $$\begin{aligned} T= \frac{-A_j+\sqrt{A_j^2-4Q_jB_j}}{2A_j}. \end{aligned}$$

**Fig. 5:**
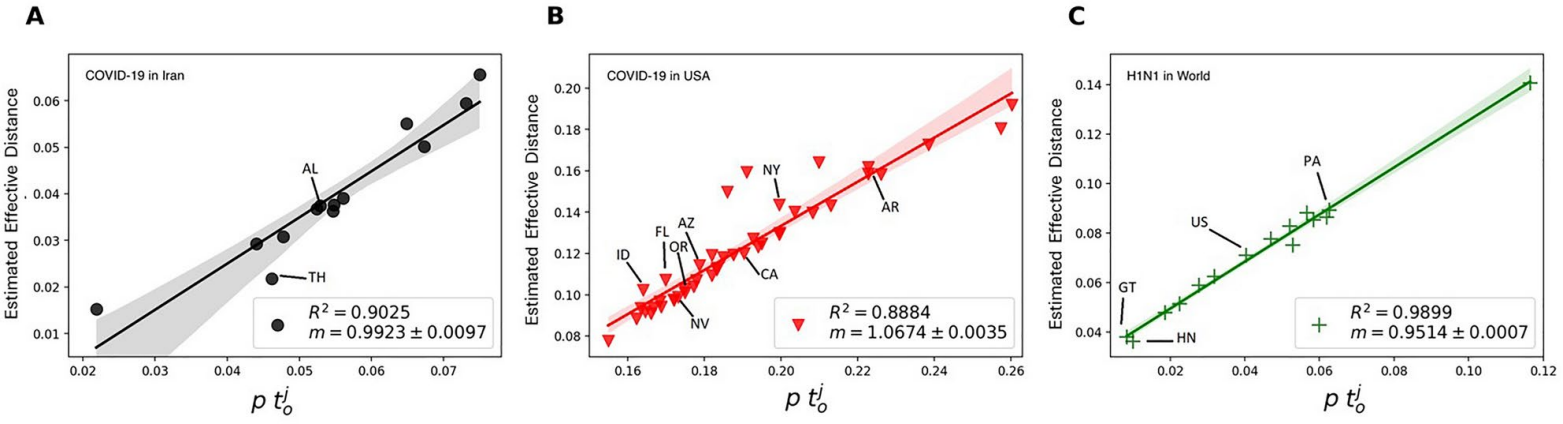
*Estimated effective distance vs overtaking time (empirical data result):* The estimated effective distance (S.M. 2.9) versus the scaled overtaking time (*pt*) is illustrated for (**A**) the COVID-19 pandemic in Iran (assuming Qom to be the source), (**B**) the COVID-19 pandemic in the US (assuming Washington to be the source), and (**C**) H1N1 pandemic in the world (2009) (assuming Mexico to be the source), We estimated the effective distance and overtaking time of the nodes, using the method in section 2.9 and the empirical data ($\vec {I^e}(t_e)$) of the pandemics. Please refer to the S. M. Section 4 for more details regarding the data.

See S.M. section 2.9 for a detailed proofs of these equations. Figure 5 shows the estimated effective distance and overtaking time for the empirical data of the COVID-19 pandemic in Iran (panel A), the US (panel B), and the H1N1 pandemic in 2009 in the meta-population of the world. As shown, there is a linear relation between effective distance and overtaking time in all instances, with the universal slope close to 1. This result demonstrates that the linear relation with a slope of one remains robust for any disease type or any size and structure of the meta-population network in which the disease spreads.

## Concluding remarks

In summary, we introduced a mathematical framework based on the SIR model for meta-population networks, incorporating inter-population mobility. We derived a compact equation (Eq. 2) that represents the time evolution of the number of infected individuals using the mathematical operator $e^{\hat{B}pt}$. We showed how different terms in the Taylor expansion of the operator represent possible transmission paths with different number of intermediary nodes. Based on this general mathematical framework and the provided data, we were able to determine where and when the outbreak began, as well as how it spread within the meta-network.

Firstly, we derived a measure indicating the contribution of each node to disease spread, whether in single-source or multi-source pandemics. Our analysis of COVID-19 revealed that Qom, Tehran, Gilan, and Mazandaran carry the greatest weight in Iran, indicating these provinces as probable sources of the pandemic. This observation aligns with the proximity of these provinces to Imam Khomeini International Airport and the relatively high volume of travel to these areas. Likewise, Washington, Michigan, New York, and California were identified as likely sources of the pandemic in the US.

Secondly, we derived an expression to find the temporal origin of a pandemic. Thus, we estimated the beginning date of the COVID-19 pandemic in Iran and the US is Feb. 8, 2020, and Feb. 12, 2020, respectively. These dates precede the officially announced start dates in both countries, suggesting that the pandemic may have begun earlier than previously thought.

Thirdly, we introduced a novel definition for effective distance and demonstrated that the effective distance of a node from the source exhibits a linear relationship with the scaled overtaking time (*pt*), characterized by a universal slope of one. Importantly, this relationship remains robust for any epidemiological parameters of the disease and characteristics of the meta-population network, such as the number of passengers and network structure. This assertion is supported by our simulation results for Iran and the US. Finally, we showed how the effective distance can be estimated only with the data of the number of infected ones in the network. We applied this method to the data from the COVID-19 pandemic in Iran and the US, as well as the 2009 H1N1 pandemic. Our analysis confirmed the existence of a linear relationship with the universal slope of one.

Combining all reported observations, our analysis underscores the following practical implications: Given that the speed of disease propagation in the network is directly proportional to travel probability, *p*, this emphasizes the crucial role of implementing travel restrictions during the early stages of a pandemic. Additionally, our findings highlight the importance of predicting more accurately when and how diseases reach the next node. This insight provides policymakers with a better understanding of the optimal strategies for implementing lockdowns or travel restrictions, thereby effectively mitigating the spread of infectious diseases.

Our work presents several theoretical implications and prospects for the research community. Firstly, unlike similar studies^13,62,66^, our definition of effective distance in this paper is directly derived from the mathematical model that describes the phenomenon, rather than relying solely on intuition or data analysis. Additionally, our analysis reveals that effective distance exhibits a universal geometric pattern, contributing to a deeper understanding of epidemic dynamics across different contexts. Secondly, our study addresses fundamental questions such as where and when pandemics begin within a coherent mathematical framework, shedding light on essential aspects of disease spread. However, our method has limitations stemming from the simplifying assumptions we made. Firstly, we utilized the SIR model for meta-population networks, which limited the scope of our study to epidemics describable by the SIR model in their early stages. However, the study can be extended by incorporating more complex epidemiological models. see S.M. Section 3 as an example. Secondly, we treated certain parameters as fixed, which may not always hold true. For instance, we assumed that the number of susceptible individuals remains constant and equal to the node’s population at the early stage of the dynamic. Additionally, we supposed that $\gamma$ is constant across nodes and that the flow matrix remains fixed over time. While these assumptions are reasonable in many cases, they may not accurately reflect reality in all scenarios. Furthermore, as demonstrated, systematic errors can arise from ignoring higher-order terms of the Taylor expansion (Eq. 4). Therefore, our algorithms and results can be enhanced by avoiding mathematical simplifications and improving data quality. Finally, we did not incorporate the effects of importation and control parameters in our model, nor did we consider various types of noise in both data sources-mobility and the number of infected cases-which could potentially influence the robustness of the results. Each of these aspects, as well as further questions such as the detection of the Big Bang for syndemic scenarios^94,95^, warrants further investigation in future studies.

## Supplementary Information

Supplementary Information.

## Data Availability

The datasets used in the current study are openly available; see the Supplementary Material, Section 4.
