# Supplementary material for: The Big Bang of an epidemic: a metapopulation approach to identify the spatiotemporal origin of contagious diseases and their universal spreading pattern
Source: Sci Rep. 2025 Feb 17;15:5809. doi: 10.1038/s41598-025-85232-7 (PMC11832755; doi:10.1038/s41598-025-85232-7)
Supplement: Supplementary file 1 — Supplementary Information. [file 41598_2025_85232_MOESM1_ESM.pdf]

The Big Bang of an epidemic: a metapopulation approach to identify the spatiotemporal origin of contagious diseases and their universal spreading pattern (Supplementary Material)

Yazdan Babazadeh Maghsoodlo[1], Amin Safaeesirat[2], and Fakhteh Ghanbarnejad[3,4,*]

[1]Department of Applied Mathematics, University of Waterloo, Waterloo, ON N2L 3G1, Canada
[2]Department of Physics, Simon Fraser University, Burnaby, Canada
[3]Potsdam Institute for Climate Impact Research (PIK), Member of the Leibniz Association, P.O. Box 601203, Potsdam, 14412, Germany
[4]School of Technology and Architecture, SRH University of Applied Sciences Heidelberg, Campus Leipzig, Prager Str. 40, Leipzig, 04317, Germany
[*]fakhteh.ghanbarnejad@gmail.com

1 General Mathematical Framework

1.1 Notation of variables and parameters

	Name	Unit	Definition
β	Transmission rate	$\frac{1}{day}$	Transmission rate in the SIR dynamics
γ	Recovery rate	$\frac{1}{day}$	Recovery rate in the SIR dynamics
R_0	Basic reproduction rate	-	The transition rate over the recovery rate
q_i	-	-	the slope of the linear part of the dynamics for node i, see sections 1.4 and 1.5.
S_i	Susceptible in the sub-population i	-	Number of susceptible individuals in node i
I_i	Infected people in node i	-	Number of infected/infectious individuals in node i
I_i^e	Reported infected individuals in node i	-	Number of infected/infectious individuals in node i reported by (empirical data)
Δ_i	MSE	-	Mean squared error between I_i and I_i^e
i_0	Total initial patients	-	The total number of patients at the beginning of the dynamic (sum over $\vec{I}(0)$).
R_i	Recovered people in a sub-population i	-	Number of recovered individuals in node i
N	Total population	-	The total population studied within the meta-population
N_i	Population of the sub-population i	-	-
n	Number of sub-populations	-	-
N_p	Total passengers population	$\frac{1}{day}$	Number of daily travelers
F_{ij}	Flow per unit time	$\frac{1}{day}$	Number of daily travelers from the node i to the node j
P_{ij}	Passenger probability per unit time	-	Probability that a traveler travels from the node i to the node j
p	$\frac{N_p}{N}$	$\frac{1}{day}$	Travel probability of an individual between sub-populations
$\hat{B}$	Time evolving operator	-	See the main text
D_{ij}	effective distance between node i and j	-	see Eq. 89
t_o^j	Overtaking time of node j	day	When the intra-population dynamics surpass the inter-population dynamics at node j, see Eq. 87

Table 1: All parameters and variables in our mathematical framework are brought here.

1.2 Definition of flow matrix and mobility probability Matrix

P_{ij}, is defined as the probability that a person travels from node i to node j. Also, p is defined as the average travel probability in the network. The *Flow Matrix* is a matrix representing the movements within the network. F_{ij} is the number of travellers from node i to j per time. So, $\sum_j F_{ij}$ is the total number of passengers exiting from the node i. Based on this definition, we make the probability matrix and the parameter p as the following:

$$P_{ij} = \frac{F_{ij}}{\sum_j F_{ij}} \tag{21}$$

$$p = \frac{\sum_j \sum_i F_{ij}}{N}, \tag{22}$$

where N is the total population of the network.

1.3 Deriving the intra population term in Eq. 3

To derive the number of infected people in node i, we have to calculate the flow of infected people in and out of this node. To calculate the number of infected people per time, who leave this node and go to other nodes, first, we can use the concept of flow and calculate the number of infected people that go to node j :

$$F_{ij}\frac{I_i}{N_i} \tag{23}$$

Then, we can sum over the index j to calculate the total number of people who leave the node i:

$$\sum_j F_{ij}\frac{I_i}{N_i}. \tag{24}$$

If we consider the travel probability in node i to be

$$p_i = \frac{\sum_j F_{ij}}{N_i}, \tag{25}$$

then we can rewrite the above equation and derive the following differential equation for the outgoing population in node i:

$$\frac{dI_i}{dt} = -p_i I_i \tag{26}$$

Now to calculate the number of infected people that enter node i, we calculate the number of infected people who travel from node j to i per time :

$$F_{ji}\frac{I_j}{N_j} \tag{27}$$

Then, if we multiply both the numerator and denominator by

$$\sum_i F_{ji} \tag{28}$$

the resulting equation is

$$\frac{F_{ji}}{\sum_i F_{ji}} \frac{\sum_i F_{ji}}{N_j} I_j. \tag{29}$$

The first term on the left side is the definition of a probability matrix, P_{ji}. So the number of infected people who travel from node j to i is

$$p_j P_{ji} I_j. \tag{30}$$

By summing up the above equation over index j, we have

$$\sum_j p_j P_{ji} I_j. \tag{31}$$

Now, the full evolution of infected people in node i is

$$\frac{dI_i}{dt} = -p_i I_i + \sum_j p_j P_{ji} I_j. \tag{32}$$

To derive the final formula, we assume the travel probability is the same among the nodes of the network and is equal to the average value of being a traveler in the whole network. Therefore

$$p_i \approx p = \frac{\sum_j \sum_i F_{ij}}{N}, \tag{33}$$

which means

$$\frac{dI_i}{dt} = -pI_i + p\sum_j P_{ji}I_j. \tag{34}$$

1.4 Deriving exponential behavior of $\vec{I}(t)$

Assuming the spread of the disease is at its early stages, we can estimate S_i with N_i and rewrite the evolution equation as

$$S_i \approx N_i \tag{35}$$

$$\frac{dI_i}{dt} = (\beta_i N_i - \gamma_i - p)I_i + \sum_j pP_{ji}I_j, \tag{36}$$

in which P^T_{ij} and q_i as,

$$P^T_{ij} = P_{ji} \tag{37}$$

$$q_i = \frac{\beta_i N_i - \gamma_i}{p}. \tag{38}$$

The equation can be rewritten as

$$\frac{dI_i}{dt} = p\sum_j (P^T_{ij} + \delta_{ij}(q_j - 1))I_j, \tag{39}$$

where δ_{ij} is the Kronecker delta and is equal to 1 when $i = j$ and zero when $i! = j$. Considering $B_{ij} = P^T_{ij} + \delta_{ij}(q_j - 1)$, the above equation can be simplified as

$$\frac{d\vec{I}}{dt} = p\hat{B}\vec{I}, \tag{40}$$

in which matrix $\hat{B}$ is

$$\hat{B} = \begin{bmatrix} q_1 - 1 & P_{21} & P_{31} & \dots \\ P_{12} & q_2 - 1 & P_{32} & \dots \\ \dots & \dots & \dots & \dots \end{bmatrix} \tag{41}$$

The solution to the above differential vector equation is

$$\vec{I}(t) = e^{\hat{B}pt}\vec{I}(0), \tag{42}$$

where, $\vec{I}(0)$ represents the initial vector of patients.

1.5 Deriving q_i in the early stage of dynamics

Assuming the spread of the disease is at early stages in the SIR model, we can estimate S_i with N_i and rewrite the evolution equation as

$$S_i \approx N_i \tag{43}$$

$$\frac{dI_i}{dt} = \beta_i N_i I_i - \gamma_i I_i, \tag{44}$$

with the solution of

$$I_i(t) = I_i(0)e^{\frac{\beta_i N_i - \gamma_i}{p}(pt)}. \tag{45}$$

The above equation can be rewritten in the simpler form of

$$I_i(t) = I_i(0)e^{q_i(pt)}. \tag{46}$$

As a result, we can expect a linear behavior if we plot $\log I_i(t)$ vs pt

$$\log I_i(t) = \log I_i(0) + q_i(pt). \tag{47}$$

The slope of this line is q_i.

1.6 $e^{\hat{B}pt}$ expansion and intermediary nodes

Eq. 42 can be expanded as

$$\vec{I}(t) = (\hat{1} + \hat{B}pt + \frac{(\hat{B}pt)^2}{2!} + ...)\vec{I}(0), \tag{48}$$

where $\hat{1}$ is the identity matrix. If we label the source node with i , and the non-source nodes with j, for node j the above equation leads to :

$$P_{ij}i_0pt + \left[\frac{i_0p^2t^2}{2}\left((P_{ij})(q_i + q_j - 2) + P_{i1}P_{1j} + P_{i2}P_{2j} + \ldots + P_{in}P_{nj}\right) + \ldots\right] \tag{49}$$

In the above equation, the term :

$$P_{i1}P_{1j} + P_{i2}P_{2j} + ...P_{in}P_{nj} = \sum_k P_{ik}P_{kj}$$

is the probability that one travels from node i to j via one of the intermediary nodes between them. We name it P^1_{ij}, therefore:

$$I_j(t) = P_{ij}i_0pt + \frac{i_0p^2t^2}{2}\left(P_{ij}(q_i + q_j - 2) + P^1_{ij}\right) + \ldots \tag{50}$$

For the source node, the evolution equation is

$$I_i(t) = i_0(1 + pt(q_i - 1)) + ... \tag{51}$$

1.7 Estimating R_0

As explained in Eq. 38, we can write q in terms of R_0 and γ and p :

$$q_i = \frac{\gamma(R_0 - 1)}{p} \tag{52}$$

Also, as shown in Section 1.5, Eq. 47, during the early stage of the dynamics, a linear relationship holds between $\log(I_i)$ and t, with a slope of $q_i \cdot p$. Consequently, it is possible to analyze the linear relationship in the early stage of the dynamics and estimate the slope and R_0. The results of this analysis indicate that for Iran, the linear fit yields an R^2 value of 0.96 and a slope of 0.141, while for the US, the linear fit yields an R^2 value of 0.96 and a slope of 0.138. Based on this, our estimate for R_0 for Iran and the US is 2.974 and 2.932, respectively.

In our study, we used $R_0 = 3$ to maintain consistency with early COVID-19 estimates that approximated R_0 around this value. This is supported by various studies on the basic reproduction number for COVID-19, which showed considerable variability but generally placed it near 3. For instance, one review reported values for ranging widely, from as low as 1.4 up to 6.49, with a mean of 3.28 across different regions and conditions [3]. Moreover, in Sec. 5 we performed a sensitivity analysis to confirm that our results remain stable despite uncertainties in the parameters used.

2 Details of Mathematical Derivations and Algorithms

2.1 Where?

Assume one has a snapshot of the disease state reported exactly t days after the beginning of the pandemic:

$$(I^e_1, I^e_2, ..., I^e_n). \tag{53}$$

The goal is to find the sources given the starting time and a snapshot of the disease.
We know that the dynamic of $\vec{I}(t)$ is:

$$\vec{I}(t) = e^{\hat{B}pt}\vec{I}(0) \tag{54}$$

If there are some nodes responsible for the spread of the disease in the network with the initial number of patient of i_{0_i}, i_{0_j}, i_{0_k}, . . . , then $\vec{I}(0)$ can be decomposed into a summation of different initial vectors, each representing a specific node. Therefore we have

$$\vec{I}(t) = e^{\hat{B}pt}(\vec{I_i}(0) + \vec{I_j}(0) + \vec{I_k}(0) + ...) \tag{55}$$

$$\vec{I}(t) = \vec{I_i}(t) + \vec{I_j}(t) + ..., \tag{56}$$

where

$$\vec{I}_i(t) = e^{\hat{B}pt}\vec{I}_i(0). \tag{57}$$

Now if we define the basis vector $\hat{i}$ to be a vector with 1 in component i and 0 in all other components, we can write:

$$\vec{I}_i(0) = i_{0_i}\hat{i} \tag{58}$$

$$\vec{I}(t) = (e^{\hat{B}pt}i_{0_i}\hat{i} + e^{\hat{B}pt}i_{0_j}\hat{j} + ...). \tag{59}$$

So if we change our basis from $\hat{i}$ into $\hat{i'}$:

$$\hat{i'} = e^{\hat{B}pt}\hat{i}, \tag{60}$$

it can be easily shown that in the linear range, these new bases are complete and orthogonal. Now, we define the "weight of a node" as

$$W_i = \frac{\hat{i'}.\vec{I}(t)}{\sum_{\hat{i'}}\hat{i'}.\vec{I}(t)}. \tag{61}$$

W_i is a number between 0 and 1 and shows the contribution of the node i at the beginning of the spread. If W_i is 1 it means that node i was responsible alone. So by calculating the defined weight for different nodes, we can understand the role and impact of each node on the spread.

2.2 Algorithmic description of Where

The following algorithm outlines the process for identifying potential sources of a disease outbreak based on empirical infection data.

Algorithm 1 Where Algorithm for Identifying Potential Sources of a Disease

Input:
    Empirical infection data vector, $\vec{I}^e(t_e)$
    Time, t_e, measured from the officially reported temporal origin
    Flow matrix, $\hat{B}$
    Parameter p representing the propagation rate
Output:
    Weights W_i^e for each node i, indicating contribution to the spread

Step 1: Define the Basis Vectors
Create an n-dimensional unit vector space where each vector represents a node
Let $\hat{i}$ be the basis vector for node i, defined as:

$$\hat{i} = (0, 0, \ldots, 1, \ldots, 0)$$

where the i-th component is 1, and all others are zero

Step 2: Transform the Basis Vectors Over Time
Evolve the basis vectors backward in time:

$$\vec{i'} = e^{\hat{B}pt}\hat{i}$$

where $e^{\hat{B}pt}$ represents the time-evolution operator

Step 3: Calculate the Contribution of Each Transformed Basis
Compute the inner product between each transformed basis vector $\vec{i'}$ and $\vec{I}^e(t_e)$

Step 4: Determine the Weights of the Nodes
Calculate the weight of each node W_i^e using:

$$W_i^e = \frac{\vec{i'} \cdot \vec{I}^e(t_e)}{\sum_{\vec{i'}}(\vec{i'} \cdot \vec{I}^e(t_e))}$$

Step 5: Interpret the Weights
if $W_i^e = 1$ **then**
    Node i is the primary source of the disease spread
else
    Multiple nodes with non-zero weights indicate potential multiple sources
end if

2.3 When

Assuming to have a snapshot of the state of the disease at a specific time written down as a vector:

$$\vec{I^e} = (I_1^e, I_2^e, ..., I_n^e) \tag{62}$$

where I_i^e represents the number of infected people in its corresponding node. n is the number of nodes in the network. We aim to determine the starting time of the disease using both the model and the snapshot. We define MSE (Mean Squared Error) as a closeness parameter for two vectors:

$$\Delta_i(t) = \frac{\sum_{j=1}^{n}(I_j - I_j^e)^2}{n} \tag{63}$$

in which i is the index of the source and I_j is the number of patients in the node j predicted by the model.

Now, we calculate the closeness parameter of the snapshot vector and the disease vector that comes out of the theory (using the first and the second term) in a determined time t:

$$\Delta_i^{\kappa=1}(t) = \frac{1}{n}((i_0(1 + (\beta N_i - \gamma - p)t) - I_i^e)^2 + \sum_{i=j,j!=i}^{n}(P_{ij}pti_0 - I_j^e)^2) \tag{64}$$

To find the minimum of the closeness parameter, we calculate the derivative of the above equation with respect to the t ($\frac{d\Delta_m(t)}{dt} = 0$)

$$t_i^* = \frac{1}{i_0}\frac{-\eta(i_0 - I_i^e) + p\sum(P_{ij}I_j^e)}{\eta^2 + p^2\sum P_{ij}^2} \tag{65}$$

in which $\eta = (\beta N_i - \gamma - p)$.

It means that the theory predicts the snapshot belongs to t days after the start of the disease. So the overtaking time would be t days before the date of the snapshot.

2.4 Algorithmic description for When

The goal of this algorithm is to estimate the true temporal origin of the outbreak, which may differ from the officially reported time.

Algorithm 2 When Algorithm for Estimating the Temporal Origin of the Outbreak

Input:

Empirical infection data vector, $\vec{I}^e(t_e)$, at reported time t_e

Estimated infection data vector, $\vec{I}(t)$, at time t

Node i identified as the source from the Where algorithm

Parameters: β_i (transmission rate), N_i (population of node i), γ_i (recovery rate), p (travel probability)

Probability matrix P_{ij}

Output: Estimated temporal origin t_i^* minimizing the difference between $\vec{I}(t)$ and $\vec{I}^e(t_e)$

Step 1: Compute the Mean Squared Error (MSE)

Calculate the MSE between the estimated and reported number of infected people:

$$\Delta_i(t) = \frac{\sum_{j=1}^{n}(I_j(t) - I_j^e(t_e))^2}{n}$$

where n is the number of nodes

Step 2: Estimate the Number of Infected People

Assume the number of infected people, $\vec{I}(t)$, originates from node i identified by the Where algorithm

Step 3: Find the Temporal Origin t_i^*

Solve for t_i^* that minimizes the MSE:

$$t_i^* = \frac{1}{i_0}\frac{-\eta(i_0 - I_i^e(t_e)) + p\sum_j(P_{ij}I_j^e(t_e))}{\eta^2 + p^2\sum_j P_{ij}^2}, \tag{66}$$

where $\eta = (\beta_i N_i - \gamma_i - p)$ and i_0 is the initial number of infected people at the source

Step 4: Include Error Adjustment

To account for error degradation, add the third term of the Taylor expansion to the error calculation (see S.M. 2.4)

2.5 Error in When algorithm

In this subsection we want to explain the way we estimated the error in When algorithm. It is crucial to note that the source of error in this algorithm is coming from the additional term in the Taylor expansion. In this case, for the source node, the dynamic is:

$$I_i(t) = C + A_i t + B_i t^2 \tag{67}$$

And for non-source nodes, the dynamic is :

$$I_j(t) = A_j t + B_j t^2 \tag{68}$$

The A,B,C coefficients are constants that are calculated via Eq.50 and Eq.51. Now we define MSE parameter:

$$\Delta = (C + A_i t + B_i t^2 - I_i^e)^2 + \sum_j (A_j t + B_j t^2 - I_j^e)^2 \tag{69}$$

which leads to the following equation:

$$\Delta = \Delta_0 + 2t^2((C - I_i^e)B_i - \sum_j (I_j^e B_j)t^2) \tag{70}$$

By rewriting the right term in the above equation:

$$\eta = 2(C - I_i^e)B_i - \sum_j (I_j^e B_j) \tag{71}$$

We are able to rewrite the MSE parameter :

$$\Delta = \Delta_0 + \eta t^2 \tag{72}$$

Now, by applying the derivative condition to the new equation :

$$\frac{d\Delta}{dt} = \frac{d\Delta_0}{dt} + 2\eta t = 0 \tag{73}$$

The condition of having zero value derivative happens in:

$$\frac{d\Delta_0}{dt} = -2\eta t \tag{74}$$

To find the specific point that the above condition is met, we use the Taylor expansion of Δ_0 function in the minimum point of the function(t^*).

$$\Delta_0(t) = \Delta_0(t^*) + \frac{1}{2}\frac{d^2\Delta_0(t)}{dt^2}(\delta t)^2 + ... \tag{75}$$

In the above equation, the first derivative term is equal to zero since the Δ_0 function is in its minimum in t^*. Then by substituting the Taylor expansion, we have :

$$\frac{\delta t}{t} = \frac{\eta}{\frac{d^2\Delta_0}{dt^2}} \tag{76}$$

2.6 Details of Effective Distance algorithm

In this part, we define the overtaking time of a disease in a specific node as the time when intra-population dynamics surpass the inter-population dynamic. The critical mathematical condition for this state for node j is :

$$(N_j\beta_j - \gamma_j)I_j = p(\sum_k P_{kj} I_k - I_j) \tag{77}$$

If we rewrite this equation, we have:

$$p(\sum_k P_{kj} I_k) - (\beta_j - \gamma_j + p)I_j = 0 \tag{78}$$

So if we define vector $\vec{A_i}$ for the j-th node:

$$\vec{A_j} = (P_{1j}, P_{2j}, ..., -(q_j + 1), ..., P_{nj}) \tag{79}$$

In which $-(q_j + 1)$ is in the j_{th} component.
Now, we can write the critical condition for node j in a simpler way:

$$\vec{A_j}.\vec{I} = 0 \tag{80}$$

If we want to calculate the overtaking time for node j, we can use the evolution equation of $\vec{I}$:

$$I(\vec{t}) = I(\vec{0}) + pt\hat{B}\vec{I_0} \tag{81}$$

If we dot product the vector $\vec{A_j}$ in both sides of the above equation and set the left side of the equation to be zero :

$$0 = \vec{A_j}.\vec{I_0} + pt_A\vec{A_j}.\hat{B}\vec{I_0} \tag{82}$$

So the overtaking time would be:

$$t_O^j = -\frac{1}{p}\frac{\vec{A_j}.\vec{I_0}}{\vec{A_j}.\hat{B}\vec{I_0}}$$

If we assume the i_{th} Node to be the source, the $\vec{I_0}$ would be :

$$\vec{I_0} = i_0(0, 0, ...1, ...0)$$

In which the 1 is in the i_{th} component. Therefore, the numerator of the overtaking time equation would be: $P_{ij}I_0$
For calculating the denominator, first we have to calculate $\hat{B}\vec{I_0}$ that would be :

$$\hat{B}\vec{I_0} = i_0(P_{i1}, P_{i2}, .., q_i - 1, ..., P_{in}) \tag{83}$$

In which the $q_i - 1$ term is in the i_{th} component. Now we have to calculate the $\vec{A_j}.\hat{B}\vec{I_0}$ that would be:

$$i_0(P^1_{i_j} - (2 + q_j - q_i)P_{i_j}) \tag{84}$$

In which $P^1_{ij} = \sum_k P_{ik}P_{kj}$
So by substituting these terms in the overtaking time equation, we have :

$$t_O^j = \frac{1}{p}\frac{1}{(2 + q_j - q_i) - \frac{P^1_{ij}}{P_{ij}}} \tag{85}$$

2.7 Algorithmic description of Effective Distance and Overtaking Time

This algorithm aims to identify simple geometric patterns in the disease spread by calculating the overtaking time and effective distance between nodes in a network.

Algorithm 3 Effective Distance and Overtaking Time Algorithm

Input:
  Parameters for each node: N_j (population), β_j (transmission rate), γ_j (recovery rate)
  Inter-population travel probability matrix P_{ij} and P^1_{ij}
  Single origin node i identified in the network
Output: Overtaking time t^j_O for each node j and effective distance D_{ij} between nodes i and j

Step 1: Define Overtaking Condition
Identify the time when intra-population spreading equals inter-population spreading:

$$(N_j\beta_j - \gamma_j)I_j = p\left(\sum_k P_{kj}I_k - I_j\right) \tag{86}$$

Step 2: Calculate Overtaking Time
Solve for the overtaking time t^j_O for node j using:

$$t^j_O = \frac{1}{p}\frac{1}{(2 + q_j - q_i) - \frac{P^1_{ij}}{P_{ij}}} \tag{87}$$

where p is the inter-population speed, and q_i, q_j are characteristics of nodes i and j

Step 3: Define Effective Distance
Define the effective distance D_{ij} based on the overtaking time:

$$D_{ij} = \frac{1}{(2 + q_j - q_i) - \frac{P^1_{ij}}{P_{ij}}} \tag{88}$$

Step 4: Establish Relationship with Overtaking Time
Ensure that D_{ij} has a linear relationship with pt^j_O:

$$D_{ij} = pt^j_O \tag{89}$$

Step 5: Simplify Effective Distance for Special Cases
if all nodes have the same value of q **then**
  The effective distance simplifies to:

$$D_{ij} = \frac{1}{2 - \frac{P^1_{ij}}{P_{ij}}} \tag{90}$$

end if

Step 6: Include Only Valid Nodes
Consider only nodes j for which $\frac{P^1_{ij}}{P_{ij}} < 2$, ensuring positive overtaking times and effective distances.

2.8 Error in Effective Distance algorithm

In this subsection, we aim to explain the way we estimated error in effective distance algorithm. The source of error here, is the additional term in the Taylor expansion. So if we keep the second term of the expansion, we have :

$$I(\vec{t}) = I_0(\vec{t}) + pt\hat{B}\vec{I_0} + \frac{1}{2}(pt)^2\hat{B}^2\vec{I_0} \tag{91}$$

Now by applying the condition of overtaking in node j:

$$0 = \vec{A_j}.\vec{I_0} + pt_j(\vec{A_j}\hat{B}\vec{I_0}) + \frac{1}{2}(pt_j)^2(\vec{A_j}.\hat{B}^2\vec{I_0}) \tag{92}$$

By rewriting time we have:

$$t_j = t^*_j + \delta t_j \tag{93}$$

In which, t^*_j is the overtaking time when we only consider the first term of the expansion. hence the equation will be in this form:

$$0 = \vec{A_j}.\vec{I_0} + pt^*_j\vec{A_j}\hat{B}\vec{I_0} + p\delta t_j\vec{A_j}\hat{B}\vec{I_0} + \frac{1}{2}(p(t^*_j + \delta t)^2\vec{A}\hat{B}^2\vec{I_0} \tag{94}$$

The first two terms in the right-hand side of the equation will cancel each other out. By simplification of the above equation the final equation for the error will be :

$$\frac{\delta t_j}{t_j^*} = \frac{-pt_j^* \vec{A}_j.\hat{B}^2 \vec{I}_0}{2(pt_j^* \vec{A}_j.\hat{B}^2 \vec{I}_0 + \vec{A}_j.\hat{B}\vec{I}_0)} \quad (95)$$

2.9 How to estimate Effective Distance without mobility data

In the previous subsection we introduced a new definition of effective distance and showed its linear relation with the overtaking time. There are two challenges when it comes to the approval of the relations with empirical data. First, The exact value of overtaking time is not known. Second, the exact value of the probability matrix is not accessible, especially after the quarantine policy in each country. In this section, we aim to bring up a novel data analysis method to overcome these challenges and confirm our theoretical achievements with empirical data. We have shown in previous sections that the temporal evolution of infected numbers is known for each node based on the general theory. Also, we know that the most accurate and accessible empirical data is the number of infected people in each node. So If we could rewrite our definition of effective distance in a way that only the number of infected people would be needed, we can achieve a new way to check our claims. Also, using official daily number of patients in different cases, we've estimated each node's overtaking time. We will show that there is a high correlation between effective distance and the estimated overtaking times.

Consider the number of infectious people versus time. Since the initial value is zero for non-source nodes, one can rewrite the equation for non-source nodes by rescaling time from t into $T = pt$:

$$I_j = A_j T + B_j T^2 \quad (96)$$

where I_j is the number of patients in the non-source node j, $A_j = P_{ij} i_0$, and $B_j = \frac{1}{2}(Pij^1 + P_{ij}(q_i + q_j - 2))$. With a simple algebra on Eq. 85 the effective distance can be rewritten according to A_j and B_j as :

$$D_{ij} = \frac{1}{q_j - \frac{B_j}{A_j}} \quad (97)$$

It is worth noting that Eq. 97 is independent of i_0, the initial number of patients, which is challenging to find at the beginning of a pandemic.

Now, we can calculate Aj and Bj by fitting a parabola to each non-source node patient data. However, there is a likely gap between official and empirical data because it takes several days for governments to identify patients at the beginning. Therefore, to fit, we've used

$$I_j = Q_j + A_j T + B_j T^2 \quad (98)$$

in which Q_j is the gap.

Moreover, to find q_j, it is enough to fit a line to the semi-log plot of patients- time, where it represents an acceptable exponential behavior, resulting from the SIR dynamics. The slop is equal to q_j.

The predicted overtaking time is the time when the number of patients is zero. After the coefficients were found, it can be calculated by solving the

$$0 = Q_j + A_j T + B_j T^2 \quad (99)$$

which leads to

$$T = \frac{-A_j + \sqrt{A_j^2 - 4Q_j B_j}}{2A_j}. \quad (100)$$

The differences in the regression of lines and number of points in this algorithm come from the accuracy of the raw data and also the mathematical condition $(\frac{P_{ij}^1}{P_{ij}} < 2)$ that constrain the presence of some nodes in our calculation in different scenarios

It is worth noting that in our formalism the linear relation between effective distance and overtaking time has y-intercept of zero and the source node has to be in (0,0) naturally. But in Figure.5 we observe that y-intercept has a non-zero value, which could be interpreted as a shift in the values of empirical overtaking times.

3 Using SEIR instead of SIR

If we consider the SEIR dynamic and its equations:

$$\frac{dE}{dt} = \beta IS - \sigma E \quad (101)$$

$$\frac{dI}{dt} = \sigma E - \gamma I \tag{102}$$

By substituting E from the first equation and putting it into the second, we will have :

$$\frac{dI}{dt} = \beta IS - \gamma I - \frac{dE}{dt} \tag{103}$$

In the first stages of the dynamic we can use two assumptions:
First, we can consider the S to be the total population which is N:

$$S \ = N \tag{104}$$

Also, we assume that the βI is small in comparison to the σE so the first equation would be in the form of :

$$\frac{dE}{dt} = -\sigma E \tag{105}$$

By using this equation we will have:

$$\frac{dI}{dt} = \beta IN - \gamma I + \sigma E(0) \tag{106}$$

Compared to the SIR model, now the solution of our model (by combining the effect of mobility) would be :

$$\vec{I(t)} = e^{\hat{B}pt}\vec{I(0)} + \sigma \vec{E(0)}t \tag{107}$$

If we expand this equation, we will have:

$$\vec{I(t)} = (\hat{I} + \hat{B}pt + ...)\vec{I(0)} + \sigma \vec{E(0)}t \tag{108}$$

We can summarize the above equation in this form :

$$\vec{I(t)} = \vec{I(0)} + (\hat{B}pt\vec{I(0)} + \sigma \vec{E(0)})t \tag{109}$$

Now by applying the Overtaking condition for node j :

$$\vec{I(t)}.\vec{A_j} = 0 \tag{110}$$

we will have :

$$0 = \vec{I(0)}.\vec{A_j} + (\hat{B}p\vec{I(0)}.\vec{A_j} + \sigma \vec{E(0)}.\vec{A_j})t \tag{111}$$

So the overtaking time will be:

$$\frac{-\vec{I(0)}.\vec{A_j}}{(\hat{B}\vec{I(0)}.\vec{A_j} + \frac{\sigma}{p}\vec{E(0)}.\vec{A_j})} = pt_o \tag{112}$$

By using Taylor expansion :

$$\frac{-\vec{I(0)}.\vec{A_j}}{(\hat{B}\vec{I(0)}.\vec{A_j} + \frac{\sigma}{p}\vec{E(0)}.\vec{A_j})} = pt_o \tag{113}$$

4 Data

In this study, we primarily utilized two different types of data: A) Snapshots of active infected cases in each subpopulation during the linear phase, some of which are visualized in Fig. 2, panels A and A', and B) Coarse-grained representations of inter-population mobility, illustrated in Fig. 2, panels B and B'. Both data types were used for Figs. 2 and 3, while only mobility data were used for Fig. 4, and only infected cases were used for Fig. 5, see section 2.9 for more details.

We obtained the number of infected people during the COVID-19 pandemic for different provinces of Iran from official reports by the Ministry of Health and Medical Education of Iran, which are available in Persian at request, and from The COVID Tracking Project at The Atlantic [1] for different states of the US. Data regarding the H1N1 pandemic was downloaded from ”www.who.int”. Abbreviations for Iranian provinces and American states are listed in Sec. 4.1.

The daily mobility data, which encompasses all forms of transportation for Iran (provided by the Basir company) and the USA [2], is averaged from March 1st to 3rd, 2020, for Iran, and from January to April 2020 for the USA.

4.1 Abbreviation

State	Abv.	State	Abv.	State	Abv.	State	Abv.
Alabama	AL	Alaska	AK	Arizona	AZ	Arkansas	AR
California	CA	Colorado	CO	Connecticut	CT	Delaware	DE
Columbia	DC	Florida	FL	Georgia	GA	Idaho	ID
Kentucky	KY	Louisiana	LA	Maine	ME	Maryland	MD
Illinois	IL	Indiana	IN	Iowa	IA	Kansas	KS
Kentucky	KY	Louisiana	LA	Maine	ME	Maryland	MD
Massachusetts	MA	Michigan	MI	Minnesota	MN	Mississippi	MS
Missouri	MO	Montana	MT	Nebraska	NE	Nevada	NV
NewHampshire	NH	New Jersey	NJ	New Mexico	NM	New York	NY
North Carolina	NC	North Dakota	ND	Ohio	OH	Oklahoma	OK
Oregon	OR	Pennsylvania	PA	Rhode Island	RI	South Carolina	SC
South Dakota	SD	Tennessee	TN	Texas	TX	Utah	UT
Vermont	VT	Virginia	VA	Washington	WA	West Virginia	WV
Wisconsin	WI	Wyoming	WY	–	–	–	–

Table 2: Abbreviation of the US states.

Province	Abv.	Province	Abv.
Ardabil	AR	Sistan-Baluchestan	SB
Ilam	IL	Golestan	GL
Khorasan-North	KS	Gilan	GI
Kurdistan-South	KJ	Yazd	YZ
Kermanshah	KM	Zanjan	ZN
Kordestan	KD	Kohgiluyeh-Boyer-Ahmad	KB
Bushehr	BU	ChaharMahaal-Bakhtiari	CB
Khorasan-Razavi	KR	Mazandaran	MZ
Khuzestan	KZ	Hormozgan	HR
Kerman	KN	Hamadan	HM
Lorestan	LR	Azerbaijan-West	AG
Qom	QM	Azerbaijan-East	AS
Semnan	SM	Fars	FR
Isfahan	ES	Qazvin	QZ
Markazi	MK	Alborz	AL
Tehran	TH		

Table 3: Abbreviation of provinces of Iran.

5 Sensitivity Analysis

These plots represent the sensitivity analysis in which γ has been changed from $\frac{1}{13}$ to $\frac{1}{20}$ and R_0 which is the basic reproductive number has been changed from 2.5 to 4.5. W_m is the weight of the node, defined in the where algorithm. By using day 5 from the official start of the pandemic we can say the 5 maximum provinces in the node power list are Qom, Tehran, Gilan, Markazi, and Alborz. These provinces stay sorted in this format for all the values of R_0 and γ. As the R_0 increases the value of error increases too. As γ decreases the value of error decreases. But by looking at 10 days we can see that the place of Tehran and Qom has been swapped. Alborz came to third place and got the previous position of Gilan. But pay attention that all these names remain reserved in the 10 days. But in 20 days Semnan appears on the list.

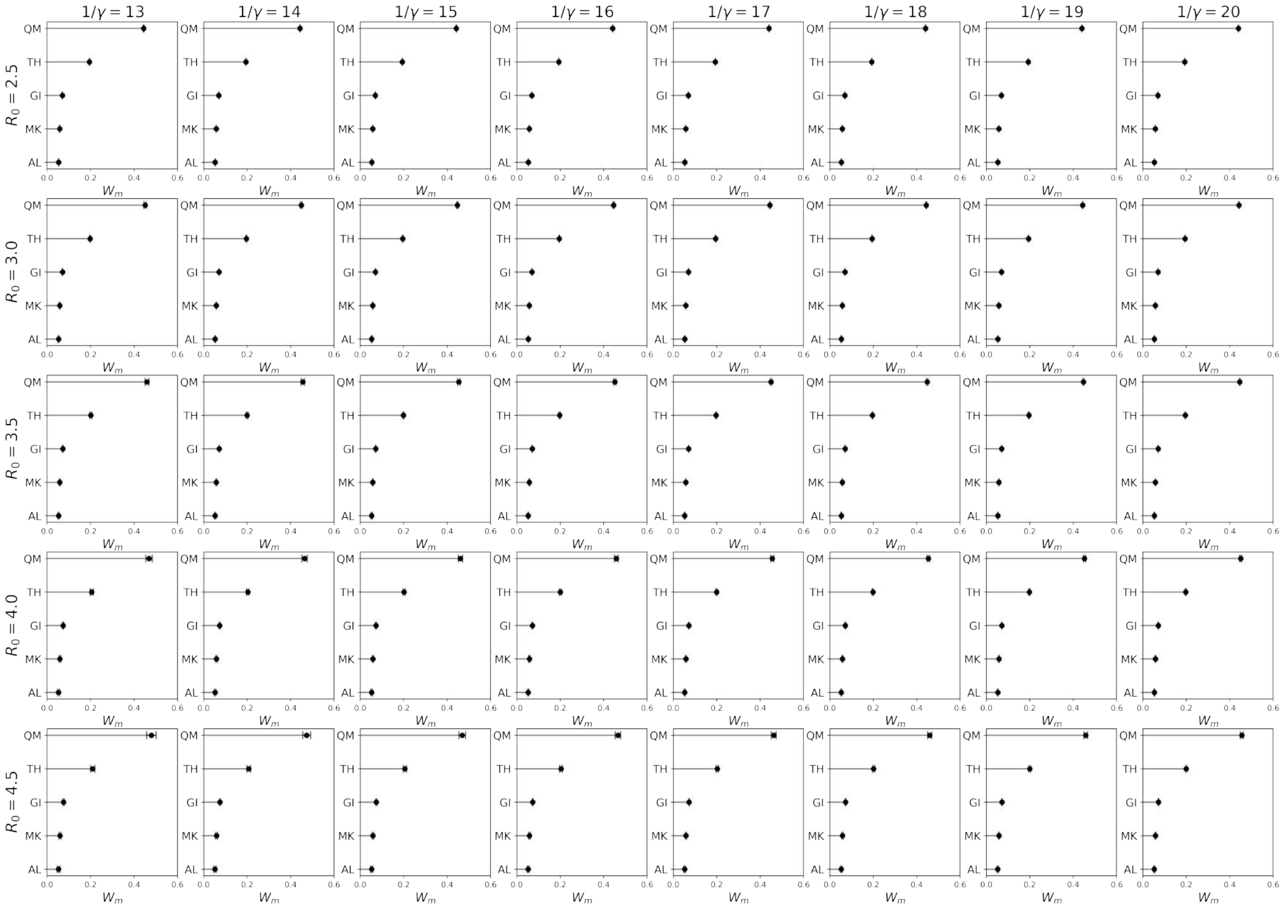

Figure 6: Calculated W_m for different provinces of Iran, and for different values of R_0 and γ, using the number of infected people in the fifth day of the COVID-19 pandemic in Iran.

References

[1] Artis Curiskis and et al. The COVID Tracking Project at The Atlantic. https://covidtracking.com/about-data, March 2021.

[2] Yuhao Kang, Song Gao, Yunlei Liang, Mingxiao Li, and Jake Kruse. Multiscale dynamic human mobility flow dataset in the u.s. during the covid-19 epidemic. *Scientific Data*, pages 1–13, 2020.

[3] Ying Liu, Albert A Gayle, Annelies Wilder-Smith, and Joacim Rocklöv. The reproductive number of covid-19 is higher compared to sars coronavirus. *Journal of travel medicine*, 2020.

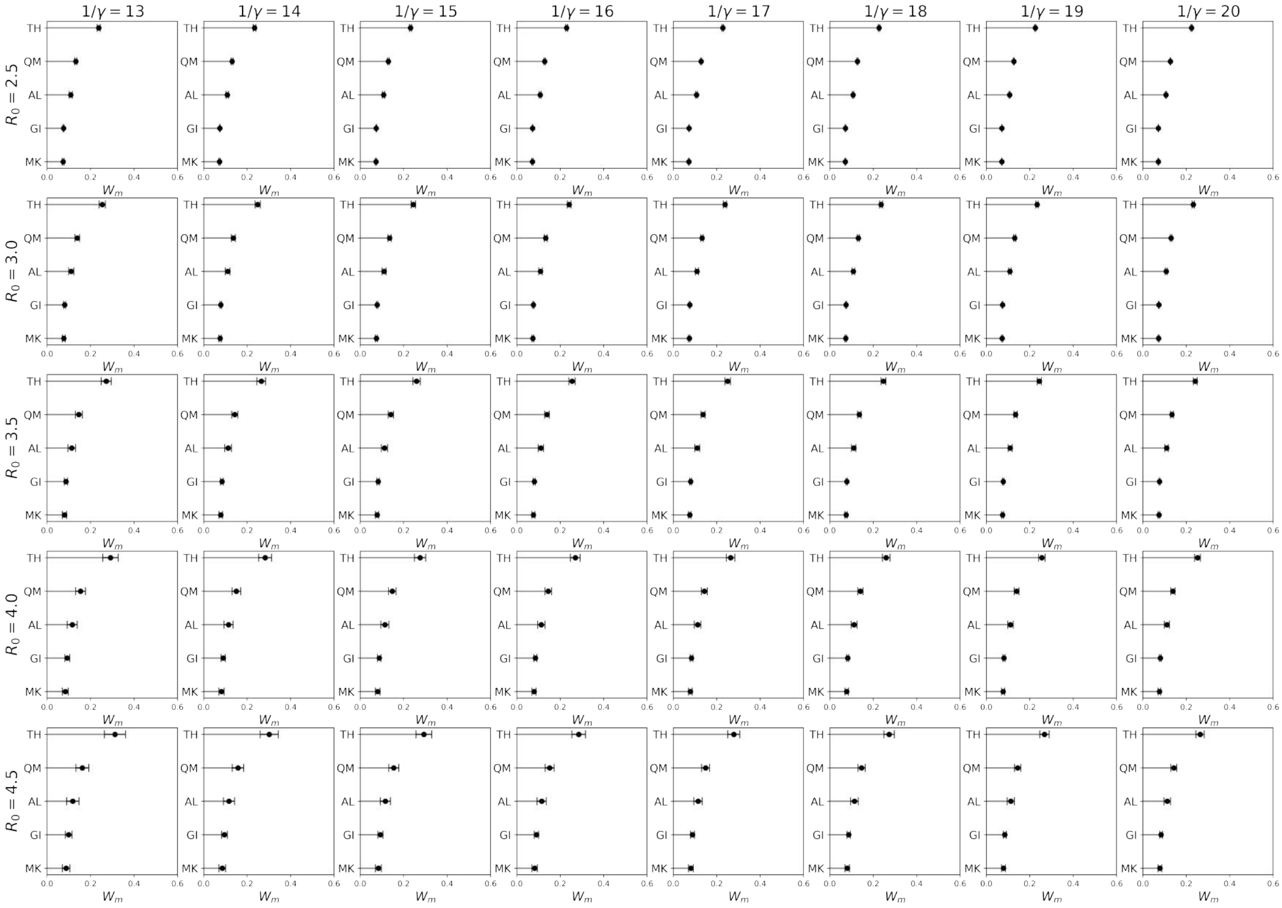

Figure 7: Calculated W_m for different provinces of Iran, and for different values of R_0 and γ, using the number of infected people in the tenth day of the COVID-19 pandemic in Iran.

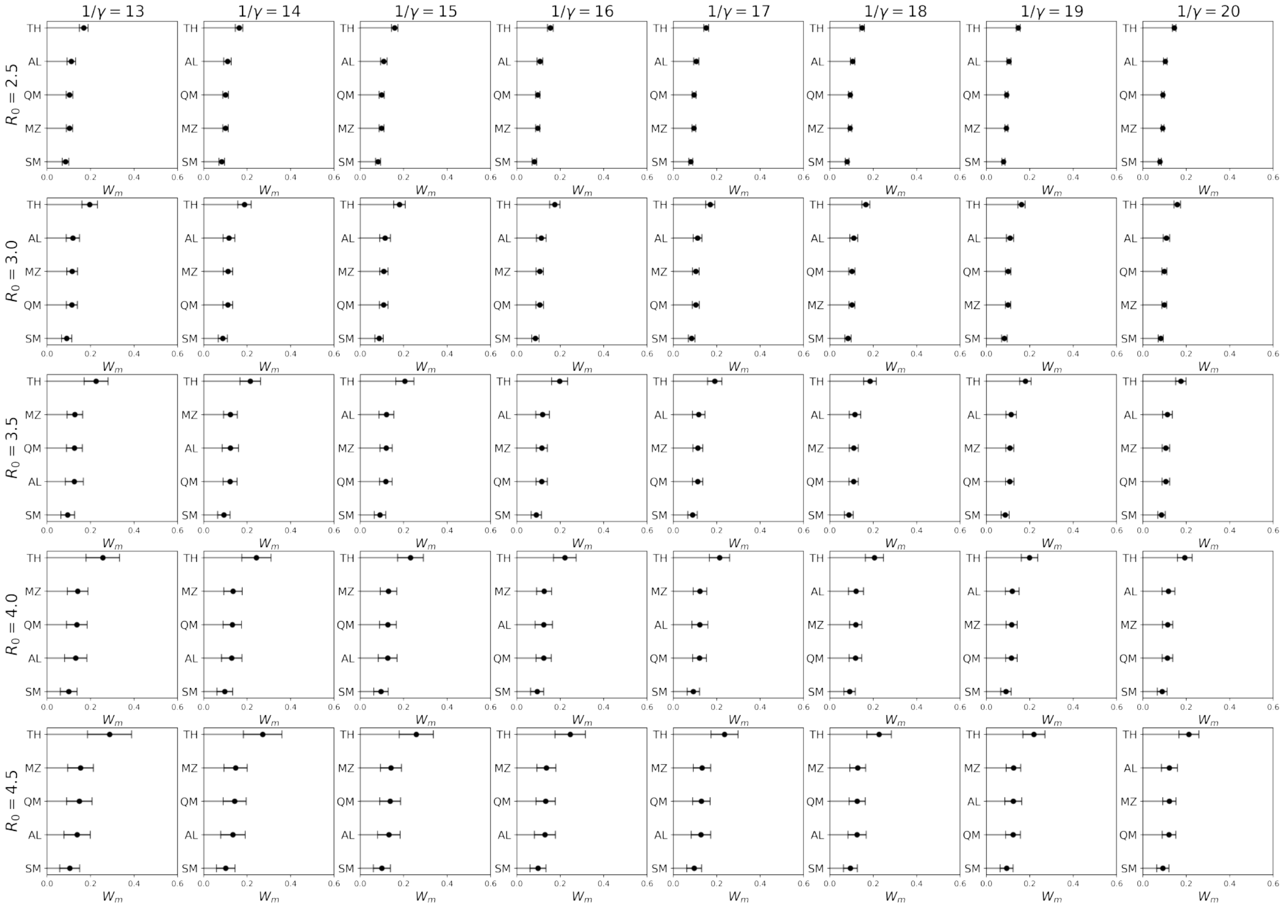

Figure 8: Calculated W_m for different provinces of Iran, and for different values of R_0 and γ, using the number of infected people in the fifteenth day of the COVID-19 pandemic in Iran.

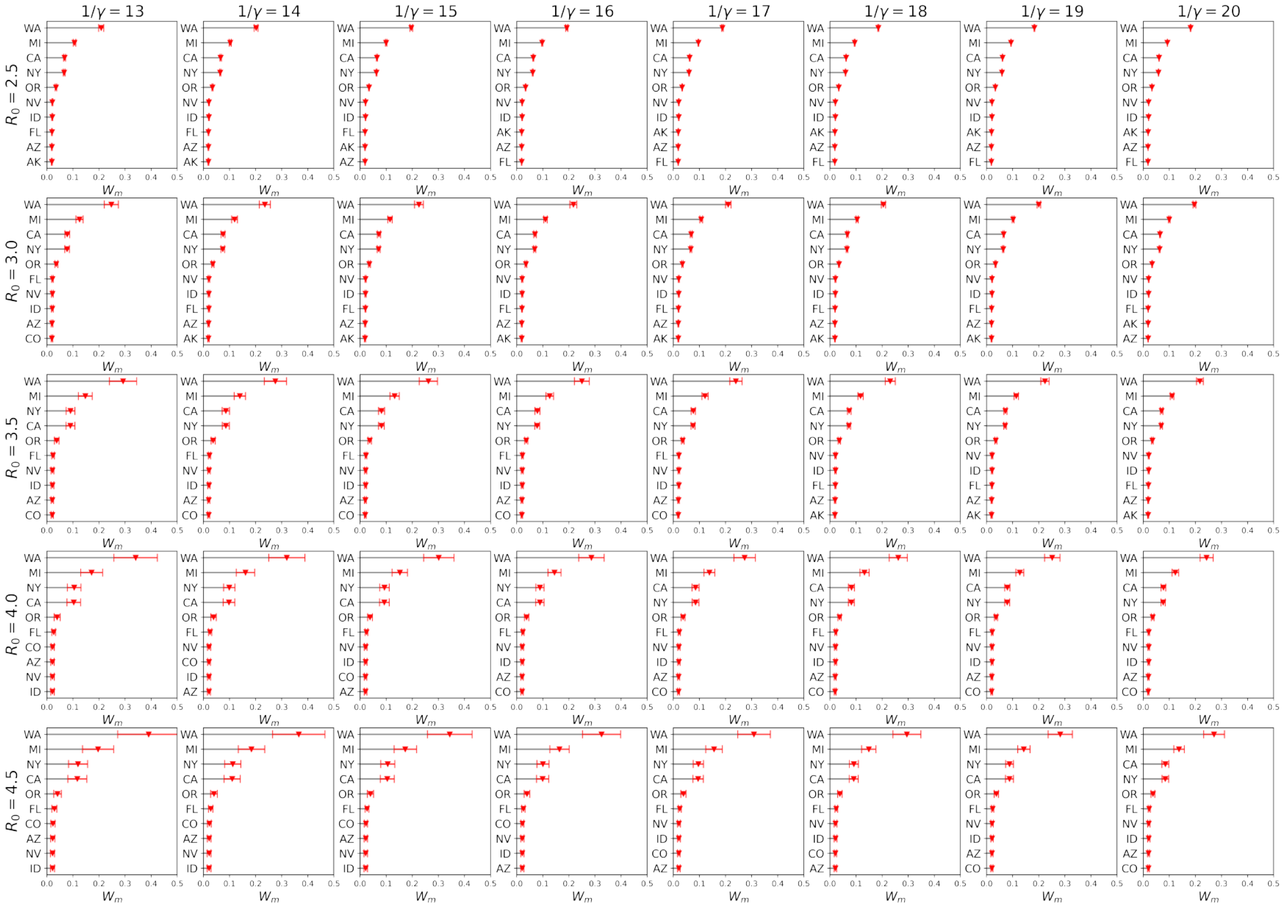

Figure 9: Calculated W_m for different states of the US, and for different values of R_0 and γ, using the number of infected individuals in day 45 of the COVID-19 pandemic in the US.

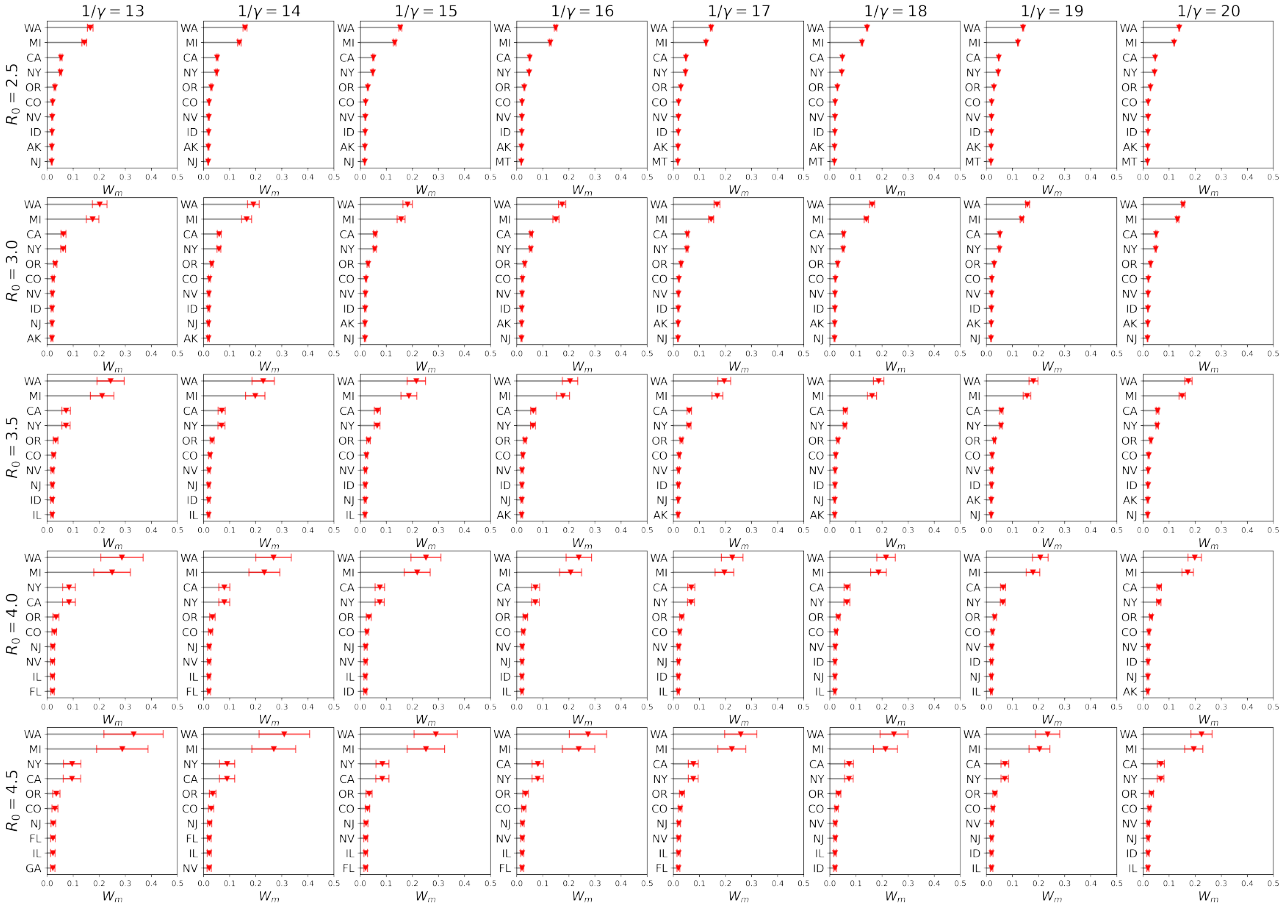

Figure 10: Calculated W_m for different states of the US, and for different values of R_0 and γ, using the number of infected individuals in day 50 of the COVID-19 pandemic in the US.

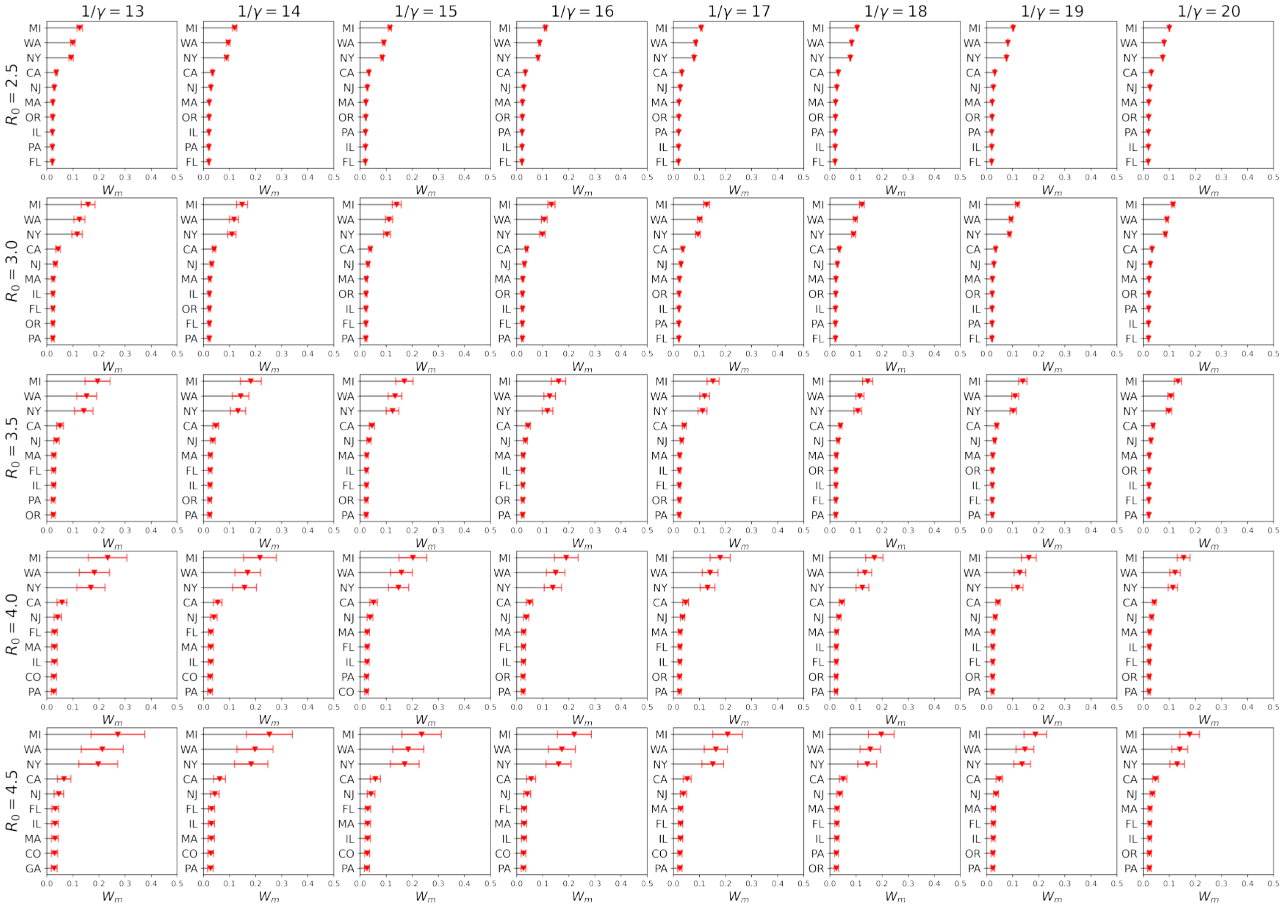

Figure 11: Calculated W_m for different states of the US, and for different values of R_0 and γ, using the number of infected individuals in day 55 of the COVID-19 pandemic in the US.
